# Genetic Determinants of Serum Testosterone Concentrations in Men

**DOI:** 10.1371/journal.pgen.1002313

**Published:** 2011-10-06

**Authors:** Claes Ohlsson, Henri Wallaschofski, Kathryn L. Lunetta, Lisette Stolk, John R. B. Perry, Annemarie Koster, Ann-Kristin Petersen, Joel Eriksson, Terho Lehtimäki, Ilpo T. Huhtaniemi, Geoffrey L. Hammond, Marcello Maggio, Andrea D. Coviello, EMAS Study Group, Luigi Ferrucci, Margit Heier, Albert Hofman, Kate L. Holliday, John-Olov Jansson, Mika Kähönen, David Karasik, Magnus K. Karlsson, Douglas P. Kiel, Yongmei Liu, Östen Ljunggren, Mattias Lorentzon, Leo-Pekka Lyytikäinen, Thomas Meitinger, Dan Mellström, David Melzer, Iva Miljkovic, Matthias Nauck, Maria Nilsson, Brenda Penninx, Stephen R. Pye, Ramachandran S. Vasan, Martin Reincke, Fernando Rivadeneira, Abdelouahid Tajar, Alexander Teumer, André G. Uitterlinden, Jagadish Ulloor, Jorma Viikari, Uwe Völker, Henry Völzke, H. Erich Wichmann, Tsung-Sheng Wu, Wei Vivian Zhuang, Elad Ziv, Frederick C. W. Wu, Olli Raitakari, Anna Eriksson, Martin Bidlingmaier, Tamara B. Harris, Anna Murray, Frank H. de Jong, Joanne M. Murabito, Shalender Bhasin, Liesbeth Vandenput, Robin Haring

**Affiliations:** 1 Center for Bone and Arthritis Research, Department of Internal Medicine, Institute of Medicine, Sahlgrenska Academy, University of Gothenburg, Gothenburg, Sweden; 2 Institute of Clinical Chemistry and Laboratory Medicine, University of Greifswald, Greifswald, Germany; 3 The National Heart, Lung, and Blood Institute's Framingham Heart Study, Framingham, Massachusetts, United States of America; 4 Department of Biostatistics, Boston University School of Public Health, Boston, Massachusetts, United States of America; 5 Department of Internal Medicine, Erasmus MC Rotterdam, Rotterdam, The Netherlands; 6 Netherlands Consortium of Healthy Ageing, Rotterdam, The Netherlands; 7 Genetics of Complex Traits, Peninsula Medical School, University of Exeter, Exeter, United Kingdom; 8 Wellcome Trust Centre for Human Genetics, University of Oxford, Oxford, United Kingdom; 9 Laboratory for Epidemiology, Demography, and Biometry, National Institute on Aging, Bethesda, Maryland, United States of America; 10 Institute of Genetic Epidemiology, Helmholtz Zentrum München, Neuherberg, Germany; 11 Department of Clinical Chemistry, University of Tampere and Tampere University Hospital, Tampere, Finland; 12 Department of Surgery and Cancer, Hammersmith Campus, Imperial College London, London, United Kingdom; 13 Child and Family Research Institute and Department of Obstetrics and Gynecology, University of British Columbia, Vancouver, Canada; 14 Departments of Internal Medicine and Biomedical Sciences, Section of Geriatrics, University of Parma, Parma, Italy; 15 Sections of General Internal Medicine, Preventive Medicine, Cardiology, and Endocrinology, Diabetes, and Nutrition, Department of Medicine, Boston University School of Medicine, Boston, Massachusetts, United States of America; 16 The European Male Ageing Study, University of Manchester, Manchester, United Kingdom; 17 Longitudinal Studies Section, Clinical Research Branch, National Institute on Aging, Baltimore, Maryland, United States of America; 18 Institute of Epidemiology II, Helmholtz Zentrum München, Neuherberg, Germany; 19 Department of Epidemiology, Erasmus MC Rotterdam, Rotterdam, The Netherlands; 20 Arthritis Research UK Epidemiology Unit, University of Manchester, Manchester Academic Health Science Centre, Manchester, United Kingdom; 21 Department of Physiology, Institute of Neuroscience and Physiology, Sahlgrenska Academy, University of Gothenburg, Gothenburg, Sweden; 22 Department of Clinical Physiology, University of Tampere and Tampere University Hospital, Tampere, Finland; 23 Hebrew SeniorLife Institute for Aging Research and Harvard Medical School, Boston, Massachusetts, United States of America; 24 Clinical and Molecular Osteoporosis Research Unit, Department of Clinical Sciences and Department of Orthopaedics, Lund University, Skane University Hospital, Malmö, Sweden; 25 Department of Epidemiology and Prevention, Wake Forest University Health Sciences, Winston-Salem, North Carolina, United States of America; 26 Department of Medical Sciences, University of Uppsala, Uppsala, Sweden; 27 Institute of Human Genetics, Technische Universität München, München, Germany; 28 Institute of Human Genetics, Helmholtz Zentrum München, Neuherberg, Germany; 29 Peninsula Medical School, University of Exeter, Exeter, United Kingdom; 30 University of Pittsburgh, Department of Epidemiology, Pittsburgh, Pennsylvania, United States of America; 31 Department of Psychiatry and EMGO Institute for Health and Care Research, VU University Medical Center, Amsterdam, The Netherlands; 32 Medizinische Klinik Innenstadt, Ludwig-Maximilians-University, München, Germany; 33 Interfaculty Institute for Genetics and Functional Genomics, University of Greifswald, Greifswald, Germany; 34 Department of Medicine, University of Turku and Turku University Hospital, Turku, Finland; 35 Institute for Community Medicine, University of Greifswald, Greifswald, Germany; 36 Institute of Medical Informatics, Biometry and Epidemiology, Ludwig-Maximilians- University, München, Germany; 37 Klinikum Großhadern, München, Germany; 38 Institute of Epidemiology I, Helmholtz Zentrum München, Neuherberg, Germany; 39 Division of General Internal Medicine, Department of Medicine, University of California San Francisco, San Francisco, California, United States of America; 40 Department of Epidemiology and Biostatistics, Institute for Human Genetics, University of California San Francisco, San Francisco, California, United States of America; 41 Andrology Research Unit, Developmental and Regenerative Biomedicine Research Group, The University of Manchester, Manchester Academic Health Science Centre, Manchester Royal Infirmary, Manchester, United Kingdom; 42 Research Centre of Applied and Preventive Cardiovascular Medicine, University of Turku, Finland; 43 Department of Clinical Physiology, Turku University Hospital, Turku, Finland; University of Michigan, United States of America

## Abstract

Testosterone concentrations in men are associated with cardiovascular morbidity, osteoporosis, and mortality and are affected by age, smoking, and obesity. Because of serum testosterone's high heritability, we performed a meta-analysis of genome-wide association data in 8,938 men from seven cohorts and followed up the genome-wide significant findings in one *in silico* (n = 871) and two *de novo* replication cohorts (n = 4,620) to identify genetic loci significantly associated with serum testosterone concentration in men. All these loci were also associated with low serum testosterone concentration defined as <300 ng/dl. Two single-nucleotide polymorphisms at the sex hormone-binding globulin *(SHBG)* locus (17p13-p12) were identified as independently associated with serum testosterone concentration (rs12150660, p = 1.2×10^−41^ and rs6258, p = 2.3×10^−22^). Subjects with ≥3 risk alleles of these variants had 6.5-fold higher risk of having low serum testosterone than subjects with no risk allele. The rs5934505 polymorphism near *FAM9B* on the X chromosome was also associated with testosterone concentrations (p = 5.6×10^−16^). The rs6258 polymorphism in exon 4 of *SHBG* affected SHBG's affinity for binding testosterone and the measured free testosterone fraction (p<0.01). Genetic variants in the *SHBG* locus and on the X chromosome are associated with a substantial variation in testosterone concentrations and increased risk of low testosterone. rs6258 is the first reported *SHBG* polymorphism, which affects testosterone binding to SHBG and the free testosterone fraction and could therefore influence the calculation of free testosterone using law-of-mass-action equation.

## Author Summary

Testosterone is the most important testicular androgen in men. Low serum testosterone concentrations are associated with cardiovascular morbidity, metabolic syndrome, type 2 diabetes mellitus, atherosclerosis, osteoporosis, sarcopenia, and increased mortality risk. Thus, there is growing evidence that serum testosterone is a valuable biomarker of men's overall health status. Studies in male twins indicate that there is a strong heritability of serum testosterone. Here we perform a large-scale genome-wide association study to examine the effects of common genetic variants on serum testosterone concentrations. By examining 14,429 men, we show that genetic variants in the sex hormone-binding globulin (*SHBG*) locus and on the X chromosome are associated with a substantial variation in serum testosterone concentrations and increased risk of low testosterone. The reported associations may now be used in order to better understand the functional background of recently identified disease associations related to low testosterone. Importantly, we identified the first known genetic variant, which affects SHBG's affinity for binding testosterone and the free testosterone fraction and could therefore influence the calculation of free testosterone. This finding suggests that individual-based SHBG-testosterone affinity constants are required depending on the genotype of this single-nucleotide polymorphism.

## Introduction

Testosterone, the most important testicular androgen in men, is largely bound to two plasma proteins. Most of the circulating testosterone (∼50–60%) is bound with high affinity to sex hormone-binding globulin (SHBG), while a smaller fraction (40–50%) is bound loosely to albumin, and 1–3% is unbound and termed free testosterone [1]. In prospective cohort studies, low serum testosterone concentrations are associated with cardiovascular morbidity, metabolic syndrome [2], [3], dyslipidemia [4], hypertension [5], type 2 diabetes mellitus [6], stroke [7], atherosclerosis [8]–[10], osteoporosis, sarcopenia, and increased mortality risk [11]–[13]. Thus, there is growing evidence that serum testosterone is a valuable biomarker of men's overall health status. Since age, body mass index (BMI), and smoking are known to affect serum testosterone concentrations [14], we used these parameters as common set of covariates in all association models. Studies in male twins indicate that there is a strong heritability of serum testosterone, with genetic factors accounting for 65% of the variation in serum testosterone [15]. However, the genetic determinants of serum testosterone and the genetic risk factors for low concentrations are poorly understood. Given the current gap in knowledge of the genetic factors that contribute to the inter-individual variability in serum testosterone concentration in men we conducted a meta-analysis of genome-wide association studies (GWAS). This two-stage meta-analysis included data from 14,429 Caucasian men from 10 independent cohorts within the Cohorts for Heart and Aging Research in Genomic Epidemiology (CHARGE) consortium. In stage one, the discovery stage, genome-wide association data from seven cohorts were meta-analyzed (n = 8,938) and all genome-wide significant findings that fulfilled the criteria described in the methods section were followed up in the three replication cohorts: one *in silico* replication cohort (n = 871) and two replication cohorts with *de novo* genotyping (n = 4,620). All association analyses of the discovery stage were conducted both with and without additional adjustment for serum SHBG concentrations. Our primary aim was to identify genetic variants reproducibly associated with serum testosterone concentrations in men, evaluated as a continuous trait. We also assessed whether the lead single-nucleotide polymorphisms (SNPs) from the continuous trait analyses had a significant influence on the risk of having low serum testosterone, defined as <300 ng/dl [16]. This level is slightly lower than that suggested recently by Wu et al. [11 nmol/l = 317 ng/dl] as one of the clinical criteria for late onset hypogonadism [17].

## Results

### Meta-analyses of genome-wide association studies for autosomal SNPs

**Figure 1:**
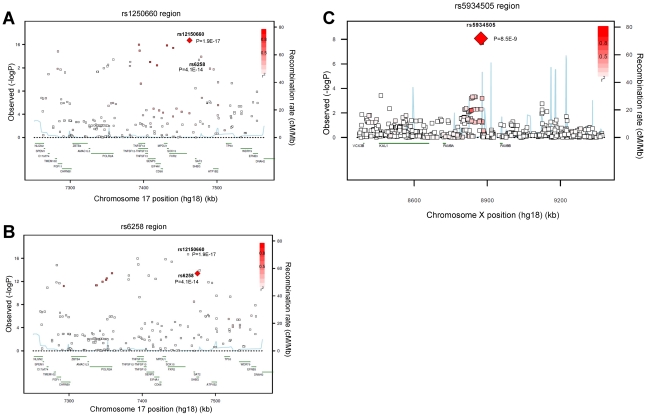
Regional association plots for single-nucleotide polymorphisms rs12150660, rs6258, and rs5934505. Regional association plot of the two independent signals on chromosome 17 with either (A) rs12150660 or (B) rs6258 indicated by red diamond to evaluate linkage with other single-nucleotide polymorphisms in the region. In addition, the association plot of the (C) rs5934505 signal on chromosome X is given. The r^2^ is based on the CEU HapMap II samples. The blue line and right hand Y axis represent CEU HapMap II based recombination rates. (A) and (B) show the top SNPs of the inverse-variance weighted discovery stage meta-analysis of untransformed serum testosterone and (C) show the top SNP of the SHBG-adjusted serum testosterone using an imputation quality filter (observed/expected variance ratio) >0.4 at the individual cohort level during meta-analysis.

We performed a GWAS of serum testosterone concentrations, investigating ∼2.5 million SNPs in 8,938 men of Caucasian ancestry, 18 to 98 years, from seven cohorts. Genome-wide significant SNPs were found in the discovery analysis at one locus on chromosome 17 (17p13-p12) using the criteria described in the methods. The strongest association was found for rs12150660 (p = 1.9×10^−17^), located 11.5 kb upstream of the major transcription start site of *sex hormone-binding globulin* (*SHBG),* with a minor allele frequency (MAF) of 23% (Table 1 [SNPs rs12150660 and rs6258], Figure 1A and Figures S1A, S2 and S3). Tests for independently associated SNPs with serum testosterone in this region revealed a second SNP, rs6258 (p = 4.1×10^−14^), which represents a missense (P→L) polymorphism located in exon 4 of *SHBG* (Table 1 [SNPs rs12150660 and rs6258], Figure 1B) and which had a MAF of 2%. Based on HapMap release 22 (CEU), the r^2^ between rs12150660 and rs6258 was 0.004. To validate the independence of these two SNPs, conditional meta-analysis of the discovery cohorts including both rs12150660 and rs6258 in an additive genetic linear model adjusted for covariates was calculated. Because the associations remained significant and mostly unchanged (rs12150660, p = 7.0×10^−14^; rs6258, p = 1.6×10^−13^), both SNPs were independently associated with serum testosterone concentrations. No additional autosomal locus fulfilled the criteria for genome-wide significance.

**Table 1:** Meta-analyses of discovery and replication cohorts.

SNPs rs12150660 and rs6258 (on chromosome 17 in *SHBG*) identified in GWAS for total testosterone														
	Discovery						Replication				Combined			
	A1/A2	FREQ*	beta	se	p	n	beta	se	p	n	beta	se	p	n
**Testosterone (ng/dl)**														
rs12150660	T/G	0.23	26.4	3.1	1.9E-17	8938	38.8	3.6	2.3E-27	5429	31.8	2.3	1.2E-41	14367
rs6258	T/C	0.02	−74.7	9.9	4.1E-14	8938	−102.9	16.3	2.9E-10	5483	−82.3	8.5	2.3E-22	14421
**SHBG (nmol/l)**														
rs12150660	T/G	0.23	3.6	0.3	3.0E-42	8366	4.4	0.4	8.5E-36	5682	3.9	0.2	2.1E-75	14048
rs6258	T/C	0.02	−6.6	0.8	1.2E-15	8366	−9.5	1.3	6.7E-14	5733	−7.4	0.7	3.5E-27	14099
**Testosterone (SHBG-adjusted)**														
rs12150660	T/G	0.23	11.1	3.0	2.5E-04	8366	11.6	3.0	9.9E-05	5414	11.3	2.1	9.0E-08	13780
rs6258	T/C	0.02	−41.8	9.4	8.2E-06	8366	−33.2	13.8	1.6E-02	5467	−39.1	7.7	4.5E-07	13833
**Calculated Free Testosterone (ng/dl)**														
rs12150660	T/G	0.23	−0.1	0.1	9.6E-02	8366	0.1	0.1	1.6E-02	5414	0.0	0.0	3.9E-01	13780
rs6258	T/C	0.02	−0.2	0.2	3.2E-01	8366	−0.5	0.3	9.0E-02	5467	−0.3	0.2	6.5E-02	13833

SNP rs5934505 (on chromosome X near *FAM9B*) identified in GWAS for SHBG-adjusted total testosterone														
	Discovery						Replication				Combined			
	A1/A2	FREQ*	beta	se	p	n	beta	se	p	n	beta	se	p	n
**Testosterone (ng/dl)**	C/T	0.26	14.1	3.2	1.1E-05	5067	27.2	6.0	5.4E-06	3816	17.0	2.8	1.7E-09	8883
**SHBG (nmol/l)**	C/T	0.26	−0.2	0.3	5.9E-01	4607	0.5	0.7	4.7E-01	4072	−0.1	0.3	8.5E-01	8679
**Testosterone (SHBG-adjusted)**	C/T	0.26	18.1	3.1	8.5E-09	4599	27.7	4.7	4.4E-09	3801	21.0	2.6	5.6E-16	8400
**Calculated Free Testosterone (ng/dl)**	C/T	0.26	0.4	0.1	4.0E-07	4607	0.6	0.1	8.7E-10	3801	0.5	0.1	6.7E-15	8408

Effects size is given per minor allele. All seven discovery cohorts (n = 8,938) were included in the GWAS of chromosomes 1–22 while only the two largest cohorts (FHS and SHIP. n = 5,067) had GWAS data available for the X chromosome. A1 = allele 1. A2 = allele 2. FREQ* = Frequency of allele 1. In the KORA cohort, testosterone was measured using plasma but the analyses after excluding KORA yielded similar results. Calculated free testosterone was calculated for all subjects with both testosterone and SHBG available by using a modified law of mass action equation. The concentrations of testosterone and SHBG and a fixed value for SHBG's dissociation constant were used in these calculations.

### Replication of autosomal hits

The associations of rs12150660 and rs6258 were confirmed in the three replication cohorts (*in silico* replication in YFS and *de novo* replication in MrOS Sweden and EMAS), demonstrating a combined p-value in the discovery and the replication cohorts of 1.2×10^−41^ and 2.3×10^−22^, respectively (Table 1 [SNPs rs12150660 and rs6258]). Both SNPs showed considerable heterogeneity of results across the studies as measured by the *I* ^2^ statistic [18]. The *I* ^2^ values for the discovery meta-analysis using the untransformed total testosterone values were 76.7% and 81.6% for rs12150660 and rs6258, respectively. The heterogeneity was reduced to 39.3% and 75.5% for rs12150660 and rs6258, respectively, by meta-analysing the z-score based untransformed total testosterone values and to 30.9% and 78.0%, respectively, by meta-analysing the inverse-normal transformed testosterone values. For rs12150660, a substantial amount of heterogeneity could be explained by phenotypic variation among the cohorts, whereas for rs6258 one cohort (InCHIANTI) showed consistent opposite effect directions in all models used. To take into account this heterogeneity, we additionally calculated a random effects model for untransformed total testosterone values. The association for rs12150660 remained genome-wide significant in the combined discovery and replication stage meta-analysis, the association for rs6258 reached genome-wide significance after excluding the InCHIANTI cohort (Table S3).

### The genetic influence on low serum testosterone concentrations

**Figure 2:**
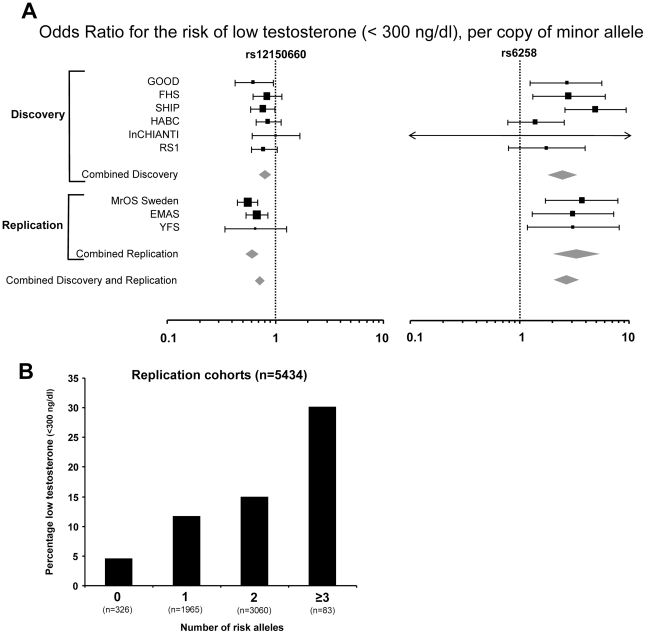
The genetic influence on low serum testosterone concentrations. (A) Odds ratio (OR) for risk of low serum testosterone concentrations (serum testosterone <300 ng/dl), per copy of minor allele. Summary estimates of the OR and their 95% confidence intervals (CI) are given. The size of the data markers is proportional to the weight (inverse of the variance) of each study. Combined discovery (n = 8,030, low serum testosterone 13%; KORA was not included as testosterone was analyzed in plasma rather than in serum, combined replication (n = 5,504, low serum testosterone 13%), and combined discovery and replication (n = 13,534, low serum testosterone 13%). (B) Percentage of men with low serum testosterone concentrations (serum testosterone <300 ng/dl), according to the number of combined risk alleles for rs12150660 (G) and rs6258 (T) in the three replication cohorts (MrOS Sweden, EMAS, and YFS). Only two individuals in the three replication cohorts had four risk alleles and therefore individuals with three and four risk alleles were pooled into one group with ≥3 risk alleles.

In Table 2, the serum testosterone concentrations according to genotype are given for the three replication cohorts. As expected, mean serum testosterone concentrations were found to be lower in men with GG than in those with TT genotype for rs12150660. Similarly, men with the CT genotype for rs6258 had lower serum testosterone concentrations than those with CC genotype. The TT genotype of rs6258 was extremely rare and only found in two subjects in the replication cohorts. The two autosomal SNPs identified by GWAS had a significant influence on the risk of having low serum testosterone (serum testosterone <300 ng/dl) in both the discovery and the replication cohorts with a combined odds ratio (OR) per minor allele of 0.72 (95% CI, 0.65 – 0.79) and 2.7 (95% CI, 2.1 – 3.5) for rs12150660 and rs6258, respectively (Figure 2A). We analyzed the combined effect of the two SNPs on the risk of having low serum testosterone concentrations according to the number of combined risk alleles for rs12150660 (G) and rs6258 (T) in the three replication cohorts (MrOS Sweden, EMAS, and YFS). The risk of having low serum testosterone concentrations increased by the number of risk alleles with an OR of 1.62 (95% CI, 1.41 – 1.86) for each risk allele (Figure S4). Low serum testosterone concentrations were 6.5-times more prevalent in men with ≥3 risk alleles (30.1% prevalence of low serum testosterone) compared to men without any risk allele (4.6% prevalence of low serum testosterone; Figure 2B).

**Table 2:** Serum sex steroids in the three replication cohorts according to rs12150660, rs6258, and rs5934505 genotype.

	SNPs identified in GWAS for total testosterone								SNP identified in GWAS for SHBG-adjusted testosterone		
	rs12150660				rs6258				rs5934505		
	GG	GT	TT	p-value	CC	CT	TT	p-value	C	T	p-value
**EMAS**	(n = 1310)	(n = 833)	(n = 152)		(n = 2261)	(n = 34)			(n = 410)	(n = 1120)	
Testosterone (ng/dl)	454±161	490±172	544±181	<0.001	474±169	358±104		<0.001	495±178	473±168	0.02
Calculated Free Testosterone (ng/dl)	8.47±2.53	8.53±2.53	8.84±2.85	0.15	8.52±2.56	8.14±2.14		0.39	9.00±2.65	8.45±2.49	<0.001
SHBG (nM)	39.6±17.1	45.2±20.4	51.6±20.8	<0.001	42.6±19.0	26.8±10.6		<0.001	42.4±20.5	42.8±18.9	0.69
**MrOS Sweden**	(n = 1317)	(n = 844)	(n = 123)		(n = 2245)	(n = 31)			(n = 530)	(n = 1765)	
Testosterone (ng/dl)	435±170	475±177	526±171	<0.001	456±174	331±125		<0.001	473±177	448±173	0.005
Calculated Free Testosterone (ng/dl)	7.98±3.07	8.30±3.16	8.75±2.99	0.005	8.16±3.08	7.59±2.72		0.31	8.54±3.27	8.03±3.03	0.001
SHBG (nM)	41.0±21.6	45.8±22.4	49.8±23.0	<0.001	43.5±22.0	24.3±12.3		<0.001	43.7±24.1	43.1±21.5	0.51
**YFS**	(n = 522)	(n = 329)	(n = 51)		(n = 852)	(n = 48)	(n = 2)				
Testosterone (ng/dl)	525±182	549±246	561±158	0.063	540±209	471±157	441±75	0.065	NA		
Calculated Free Testosterone (ng/dl)	11.89±5.30	12.30±8.92	11.57±2.46	0.71	12.04±6.90	11.80±3.42	11.55±1.23	0.80	NA		
SHBG (nM)	30.0±11.7	31.3±11.9	35.2±13.1	0.007	31.2±12.0	23.0±8.1	20.5±4.0	<0.001	NA		

NA = not available. Free testosterone was calculated for all subjects with both testosterone and SHBG available by using a modified law of mass action equation. The concentrations of testosterone and SHBG and a fixed value for SHBG's dissociation constant were used in these calculations.

### The role of SHBG in the observed associations

As SNP rs12150660 is located 11.5 kb upstream of *SHBG* and SNP rs6258 is non-synonymous and located in exon 4 of *SHBG*, we evaluated the influence of these polymorphisms on SHBG concentrations. Both of these polymorphisms demonstrated a significant association with SHBG concentrations in both the discovery and replication cohorts (Table 1 [SNPs rs12150660 and rs6258]). However, even after adjusting for SHBG concentrations, the associations between these two SNPs and serum testosterone concentrations were still significant (p = 9.0×10^−8^ for rs12150660 and p = 4.5×10^−7^ for rs6258). Free testosterone calculated using law-of-mass-action equation was not associated with either of the two polymorphisms (Table 1 [SNPs rs12150660 and rs6258]). As serum testosterone and SHBG are highly correlated (e.g., in MrOS Sweden r_s_ = 0.53), variations in SHBG concentration might have influenced the observed associations of serum testosterone with other non-SHBG-related loci. Therefore, we performed an additional SHBG-adjusted genome-wide meta-analysis among the discovery cohorts, wherein none of the non-SHBG-related autosomal SNPs reached genome-wide significance (Figure S1B).

### The rs6258 polymorphism affects SHBG binding affinity for testosterone and the measured free testosterone fraction

**Figure 3:**
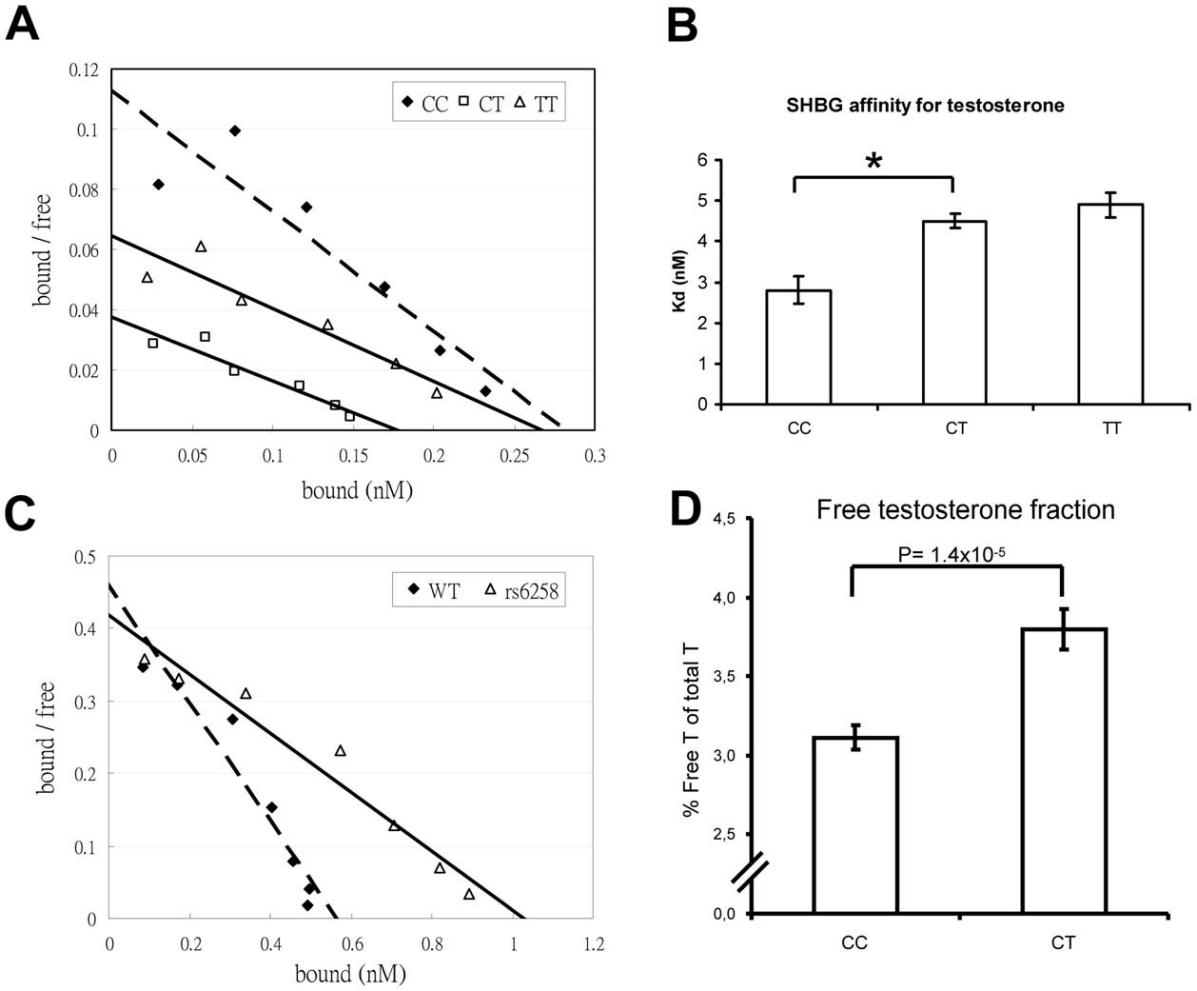
SHBG affinity for testosterone. (A and B) Scatchard plots of SHBG binding affinity for testosterone in serum samples according to rs6258 genotype. (A) Representative Scatchard plots of serum SHBG binding to [^3^H]testosterone. Serum from individuals homozygous for the wild-type SHBG allele (CC dashed line) or the rs6258 SNP (TT, solid line), or heterozygous for these alleles (CT, solid line). (B) Dissociation constant (Kd) of serum SHBG according to rs6258 genotype (CC, n = 4 subjects; CT, n = 4 subjects; TT [rare variant] n = 1 and the variation for the TT subject is derived from three separate analyses). (*) p = 0.001. Values are means ± SEM. (C) Representative Scatchard plots of recombinant SHBG binding to [^3^H]testosterone. Recombinant wild type ( = WT, C genotype; dashed line) or rs6258 (T genotype; solid line) SHBG expressed by CHO cells was diluted 1∶10 and subjected to Scatchard analysis, as in panel A. (D) Free testosterone fraction in serum measured by an equilibrium dialysis method according to rs6258 genotype (CC, n = 87 subjects; CT, n = 32 subjects). Values are means ± SEM.

As rs6258 is non-synonymous (P156L) and located in exon 4 of *SHBG,* we evaluated the serum SHBG steroid-binding capacity of the different rs6258 genotypes. As shown in Figure S5, serum SHBG from CT but not CC subjects had a lower steroid-binding capacity than expected from values obtained by an SHBG immunoassay (p = 0.003). Therefore, we analyzed the SHBG affinity for testosterone using Scatchard plots of SHBG in serum of men with the rs6258 genotype (Figure 3A), and revealed (Figure 3B) a higher mean dissociation constant (Kd) indicative of a lower affinity in CT (Kd = 4.5 nM) and TT (Kd = 4.9 nM) individuals than in CC individuals (Kd = 2.8 nM). Recombinant SHBG corresponding to the T genotype demonstrated a higher dissociation constant (lower affinity) compared with recombinant SHBG corresponding to the C genotype (T genotype Kd 2.5 nM; C genotype Kd 1.2 nM, Figure 3C). In addition, the free testosterone fraction measured by an equilibrium dialysis method was 22% higher (p = 1.4×10^−5^) in serum from CT subjects than in serum from CC subjects (Figure 3D).

### X chromosome analyses

Imputed values for X chromosome-located SNPs were available for the two larger discovery cohorts (SHIP and FHS; n = 5,067). We performed meta-analyses of imputed X chromosome SNPs for serum testosterone concentrations both with and without SHBG adjustment, revealing one genome-wide significant association for SNP rs5934505 (p = 8.5×10^−9^) in the SHBG-adjusted model (Table 1 [SNP rs5934505] and Figures S1B and S3). This SNP was confirmed in the two replication cohorts with *de novo* genotyping (MrOS Sweden p = 3.6×10^−3^; EMAS p = 1.5×10^−7^). Meta-analysis of discovery and replication cohorts resulted in a combined p-value of 5.6×10^−16^. The rs5934505 SNP is located in a CNV-insertion area (Xp22), 145 kb upstream of the *family with sequence similarity 9, member A (FAM9A*) and 79 kb downstream of the *family with sequence similarity 9, member B* (*FAM9B*) (Figure 1C). In addition, rs5934505 is located 214 kb upstream of Kallmann syndrome 1 sequence (*KAL1*). SNP rs5934505 was associated with serum testosterone without SHBG-adjustment (combined p-value of 1.7×10^−9^) and with free testosterone (combined p-value of 6.7×10^−15^), but not with SHBG (Table 1 [SNP rs5934505]). The mean serum testosterone and calculated free testosterone but not SHBG concentrations were lower in men with T genotype than in those with C genotype for rs5934505 (Table 2).

## Discussion

This GWAS revealed novel genetic variants that significantly affect circulating testosterone concentrations in men. The presence of three or more risk alleles for the two polymorphisms in the *SHBG* loci resulted in markedly decreased testosterone concentrations compared to men with two or less risk alleles. Importantly, one of the identified genetic variations was associated with an alteration in SHBG's binding affinity for testosterone and the measured free testosterone fraction. In addition, we identified a locus on the X chromosome influencing serum testosterone concentrations. The genetic contribution of the polymorphisms to testosterone concentrations reported here is substantial; as a reference for comparison, the effect of these polymorphisms on testosterone concentrations in men is similar or greater than that for known risk factors such as age, smoking, and BMI [19], [20].

These findings improve our understanding of the genetic factors that affect serum testosterone concentrations and contribute to the variation in testosterone concentrations in men. These polymorphisms may assist in the identification of men at risk of low serum testosterone, although the clinical usefulness of these findings remains to be established. As rs12150660 and rs6258 were strongly associated with SHBG concentrations, both SNPs may at least partly affect total testosterone concentrations by modulating SHBG concentrations. Our findings that rs6258 substantially affects SHBG binding affinity and the measured free testosterone fraction raise questions about the use of a single consensus value for SHBG's dissociation constant in the law of mass action equations used to calculate free testosterone concentrations. As emphasized by the Endocrine Society's expert panel on androgen deficiency syndromes, low testosterone concentrations alone should not necessarily be viewed as evidence of androgen deficiency [16]. Whether rs593405 near the *FAM9B* and *KAL1* genes on Xp22 renders men susceptible to the increased risk of androgen deficiency remains to be determined. Further studies are required to determine the impact of these genetic variations on sex steroid-related disorders, including osteoporosis, cardiovascular diseases, prostate cancer, and male infertility [21].

Our studies add to the evidence that genetic variations within the *SHBG* gene may explain some of the inter-individual differences in SHBG concentrations. Our finding that SNP rs6258 results in the production of an *SHBG* variant with reduced affinity for testosterone provides an explanation for the association between rs6258 and low serum testosterone concentrations. This is the first described genetic variant associated with altered SHBG binding for testosterone. As rs6258 is non-synonymous (P156L), located in exon 4 of *SHBG* and associated with altered SHBG binding for testosterone and free testosterone fraction, rs6258 is likely a functional polymorphism with impact on testosterone binding to SHBG as well as testosterone bioavailability and action at target tissue level.

The SNP rs12150660 that is strongly associated with testosterone concentrations is located 11.5 kb upstream of the coding sequence for SHBG mRNA production in the liver. However, it still resides within the human *SHBG* locus because several other alternative exon 1 sequences are located up to ∼13 kb upstream of the exon 1 sequence that encodes the secretion signal polypeptide of the SHBG precursor in the liver [22]. There are no obvious nuclear protein binding sites within the sequences spanning SNP rs12150660, and it remains to be determined whether this SNP disrupts a cis-element that directly influences *SHBG* transcription. We have found that rs12150660 is in strong LD (r^2^ = 0.89) with another common SNP (rs1799941) in the *SHBG* proximal promoter that was shown to be associated with serum SHBG concentrations [23]–[25]. Thus, it is highly likely that only one of these polymorphisms is actually functional and therefore both SNPs represent the same signal. It should also be noted that rs1799941 is linked to the number of TAAAA repeats within an Alu sequence upstream of *SHBG* promoter [26] and that the rs1799941 (A allele) is linked with the presence of six TAAAA repeats in this location which has been reported to be associated with higher SHBG concentrations [27]. In addition, while there does not appear to be any putative transcriptional factor binding sites with the sequence comprising rs12150660, it remains to be determined whether rs12150660 or these other associated SNPs in the *SHBG* gene are functionally important or simply represent proxies of SHBG and testosterone concentrations in men.

Our meta-analyses of imputed X chromosome SNPs revealed one genome-wide significant association for SNP rs5934505, located in a CNV-insertion area (Xp22), 145 kb upstream of *family with sequence similarity 9, member A* (*FAM9A*) and 79 kb downstream of *family with sequence similarity 9, member B* (*FAM9B*). Both genes, *FAM9A* and *FAM9B*, are expressed exclusively in the testis [28] and described here for the first time to be associated with total as well as free testosterone concentrations. rs5934505 is located 214 kb upstream of Kallmann syndrome 1 sequence (*KAL1*). Although the Kallmann syndrome, a type of hypogonadotropic hypogonadism associated with anosmia and other congenital anomalies, has been linked to mutations in the *KAL1* gene on the X chromosome, only 11–14% of Caucasian patients with hypogonadotropic hypogonadism have detectable *KAL1* mutations [29], reflecting the considerable genetic heterogeneity of this syndrome.

The strengths of our study include a discovery sample size of 8,938 men, which allowed us at the threshold α = 5×10^−8^,a 90% power to detect SNPs accounting for 0.5% of the total variance in serum testosterone concentrations, and 99% power to detect SNPs accounting for 1% of the total variance. The SNPs rs12150660, rs6258, and rs5934505 explained 2.3%, 0.9%, and 0.6%, respectively, of the variance in serum testosterone concentrations when evaluated in the MrOS Sweden replication cohort. Future meta-analyses including larger samples will probably reveal additional loci associated with serum testosterone. Further research into the functional significance of these variants will be needed to enable the translation of these findings into the mechanisms of sex steroid-related diseases and strategies for risk assessment. As the causal or etiological role of these polymorphisms in the genesis of low testosterone has not been established, the reported polymorphisms associated with low serum testosterone concentration may be viewed currently as risk markers rather than causal risk factors.

In conclusion, genetic variants in the *SHBG* locus and on the X chromosome are associated with a substantial variation in testosterone concentrations and increased risk of low testosterone in men. Further studies are needed to determine the impact of these genetic variations on sex hormone-related disorders. rs6258 is the first reported *SHBG* polymorphism, which affects testosterone binding to SHBG and the free testosterone fraction and could therefore influence the calculation of free testosterone using law-of-mass-action equation.

## Methods

### Study samples and genotyping

The discovery stage of the GWAS included 8,938 Caucasian men of European descent drawn from seven epidemiological cohorts: the Framingham Heart Study (FHS), the Study of Health in Pomerania (SHIP), the Gothenburg Osteoporosis and Obesity Determinants (GOOD) study, the Cooperative Health Research in the Region of Augsburg (KORA) study, the Health, Aging and Body Composition (HEALTH ABC) study, the Rotterdam Study (RS1), and the Invecchiare in Chianti (InCHIANTI) (Table S1). The replication stage consisted of 4,620 men from two epidemiological cohorts (the European Male Ageing Study [EMAS] and the Osteoporotic Fractures in Men [MrOS] Sweden study) for *de novo* genotyping of the top SNPs and one additional cohort (the Young Finns Study, [YFS, n = 871]) with genome-wide association data available and joining the study after stage one was finished for *in silico* replication (Table S2).

Exclusion criteria included chemical or surgical castration and/or medications affecting sex hormones such as steroid 5-alpha reductase inhibitors, and sex hormone antagonists. All studies were approved by local ethics committees and all participants provided written informed consent. Characteristics of the study samples and detailed descriptions of the participating cohorts, genotyping methods, quality control, and imputation procedures are provided in Text S1.

### Genotyping and statistical analyses

Altogether, ∼2.5 million SNPs, imputed using the HapMapII CEU population, were tested for association with serum testosterone in the discovery stage. Genome-wide association analyses using an additive genetic linear regression model adjusted for age, BMI, and current smoking were conducted twice within each of the discovery cohorts using serum testosterone expressed as ng/dl, as well as inverse-normal transformed serum testosterone as outcomes.

To examine the robustness of the discovery results and to reduce the risk of spurious associations due to possible testosterone measurement heterogeneity between the individual cohorts, three different types of meta-analyses were performed in the discovery stage: 1) an inverse-variance weighted fixed effect model; 2) a z-score based analysis of the untransformed serum testosterone concentrations; and 3) a z-score based meta-analysis of the inverse-normal transformed values. Model 1) was used as main analysis since it allowed the computation of effect estimates, whereas the other two analysis models were used for verification and quality control checks of the main findings. All meta-analyses were performed using METAL (www.sph.umich.edu/csg/abecasis/metal/). The random effects model of the two *SHBG* locus SNPs was calculated using the R-package *metafor* (www.r-project.org). Imputed genotypes were analyzed in all cohorts taking the genotype uncertainties into account. Genomic control was applied to each individual cohort's results and to the discovery stage meta-analysis to correct p-values for potential effects of mild population stratification. The estimated genomic control lambda was low for both the individual cohorts (range of λ_GC_: 1.00–1.07) and the meta-analyses (range of λ_GC_: 1.01–1.02), suggesting little residual confounding due to population stratification (Figure S2).

To reduce the variance on serum testosterone induced by SHBG concentration, the GWAS included a genome-wide test for association of untransformed serum testosterone concentrations adjusted for age, BMI, current smoking, SHBG and SHBG^2^ concentrations, again using both an inverse-variance weighted fixed effect as main analysis and a z-score based meta-analysis for quality control purposes.

A threshold of p<5×10^−8^ was established *a priori* as the level for genome-wide significance in the discovery analyses [30]. SNPs that reached genome-wide significance in the inverse-variance weighted meta-analysis of untransformed serum testosterone concentrations with or without adjustment for SHBG and which had association results in at least five of the seven cohorts (for chr X: two cohorts with data available) were selected for further analyses. Notably, all autosomal SNPs that fulfilled these criteria also reached genome-wide significance in the other two types of meta-analyses. From these SNPs, all independent SNPs were taken to the replication stage.

We also assessed whether the lead SNPs from the continuous trait analyses were associated with low serum testosterone concentration (defined as <300 ng/dl [16]; this level is slightly lower than that suggested recently by Wu et al [11 nmol/l = 317 ng/dl] [17]) by binary logistic regression including the same covariates in the model used for the main analysis and meta-analyzing the within-cohort results using inverse-variance weighted fixed-effect model. The KORA cohort was not included in the meta-analyses of low serum testosterone as testosterone was measured using plasma in this cohort.

We determined the number of low serum testosterone concentration risk alleles (0 to 4) for the two lead SNPs of the *SHBG* locus in each individual and assessed the risk of low serum testosterone concentrations in the three replication cohorts (MrOS Sweden, EMAS, and YFS) using a trend test. Since only two subjects in the replication cohorts had four risk alleles, individuals having three and four risk alleles were grouped into one category to obtain more reliable effect estimates during the subsequent analyses. Details of test for independence, SHBG related analysis of the top SNPs and quality control steps performed can be found in Text S1.

### Sex hormone measurements

Methods for the measurement of serum testosterone and SHBG are given in Text S1. Calculated free testosterone was for all subjects with both testosterone and SHBG available (n = 13833; Table 1 and Table 2) calculated by using a modified law of mass action equation, as described by Mazer [31]. The concentrations of testosterone and SHBG and a fixed value for SHBG's dissociation constant were used in these calculations.

### Free testosterone fraction

Free testosterone fraction was measured by an equilibrium dialysis method in 87 subjects with the CC genotype and 32 subjects with the CT genotype of rs6258 (Figure 3D) [32]. Detailed description of the free testosterone fraction measurements is provided in Text S1.

### Sex hormone-binding globulin assays

In experiments evaluating SHBG binding capacity, serum SHBG concentrations were determined by two-site immunofluorometric assay (PerkinElmer Life Sciences, Turku, Finland) [33], or by a steroid-binding capacity assay [34]. For steroid-binding assays, serum samples were pre-incubated with dextran-coated charcoal (DCC) to remove endogenous steroids, prior to incubation with either [^3^H]5α-dihydrotestosterone ([^3^H]DHT; specific activity 50 Ci/mmol) or [^3^H]testosterone (specific activity 40 Ci/mmol), bound from free [^3^H]steroid were separated using DCC as the separation reagent [34]. The steroid-binding properties of SHBG in diluted serum samples or tissue culture medium were determined by Scatchard analysis [34]. For the expression of SHBG protein, wild type (corresponding to the C genotype of rs6258) and rs6258 (corresponding to the T genotype of rs6258) SHBG cDNAs in the pRC/CMV expression vector were transfected into CHO cells, and G418 was used for selection of stably transfected cells. At near confluence, cells were washed with PBS and cultured in serum-free SFM4CHO medium (Thermo Scientific HyClone, Logan, UT) for four days before the SHBG-containing medium was harvested.

## Supporting Information

Figure S1 Manhattan plots giving genome-wide –log_10_ p-value according to chromosomal location for inverse-variance weighted meta-analysis of untransformed serum testosterone (A) and SHBG-adjusted serum testosterone (B) using an imputation quality filter (observed/expected variance ratio) >0.4 at the individual cohort level during meta-analysis. All seven discovery cohorts (n = 8,938) were included in the GWAS of chromosomes 1–22 while only the two largest cohorts (FHS and SHIP, n = 5,067) had GWAS data available for the X chromosome.
(PDF)

Figure S2 Quantile-quantile plot of the genome-wide association results of the inverse-variance weighted meta-analysis of untransformed serum testosterone including all SNPs (black) and after removal of the SNPs of the *SHBG* locus (blue).
(PDF)

Figure S3 Associations for (A) rs12150660 and (B) rs6258 with testosterone and for (C) rs5934505 with SHBG-adjusted testosterone. Effects sizes are given per minor allele. Beta estimates and their 95% confidence intervals are given. The size of the data markers is proportional to the weight (inverse of the variance) of each study.
(PDF)

Figure S4 Risk of low serum testosterone concentrations (serum testosterone <300 ng/dl), according to the number of combined risk alleles for rs12150660 (G = risk allele) and rs6258 (T = risk allele) in the three replication cohorts (MrOS Sweden, EMAS, and YFS). Bars indicate 95% confidence intervals. Only two individuals in the three replication cohorts had four risk alleles and therefore individuals with three and four risk alleles were pooled into one group with ≥3 risk alleles. Two risk allele counts were used as reference, since this is the most prevalent amount among the cohorts.
(PDF)

Figure S5 Subjects heterozygous for the *SHBG* allele containing an rs6258 SNP have lower serum SHBG steroid-binding capacity (Y-axis) when compared to the concentrations of SHBG measured by immunoassay (X-axis). Serum SHBG concentrations from 10 individuals homozygous for the wild type *SHBG* allele (CC, dashed regression line r^2^ = 0.872) or heterozygous for the rs6258 variant *SHBG* allele (CT, solid regression line r^2^ = 0.866) were measured by a time-resolved immunofluorometric assay[33], and a steroid-binding capacity assay using [^3^H]DHT as the labelled ligand.[34]
(PDF)

Table S1 Characteristics of 14,429 men from 10 cohorts included in the genome-wide association study meta-analysis.
(PDF)

Table S2 Additional genotyping information for the 10 cohorts included in the genome-wide association study meta-analysis.
(PDF)

Table S3 Meta Analysis of untransformed total testosterone using Random Effect Model.
(PDF)

Text S1 Supplemental methods.
(DOC)
