# Supplementary figures and images for: Genetic Determinants of Serum Testosterone Concentrations in Men
Source: PLoS Genet. 2011 Oct 6;7(10):e1002313. doi: 10.1371/journal.pgen.1002313 (PMC3188559; doi:10.1371/journal.pgen.1002313)

**Figure S1**

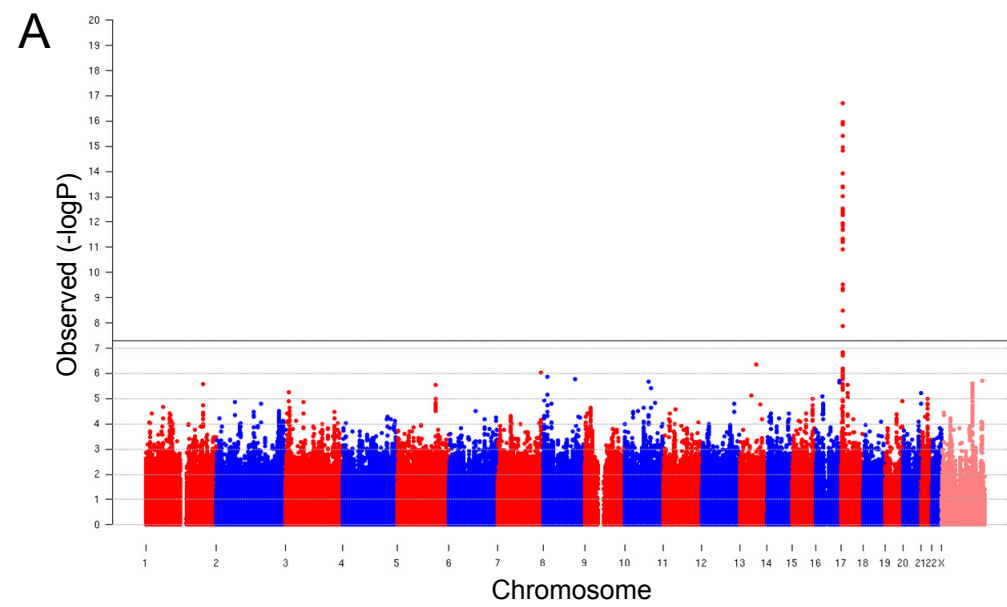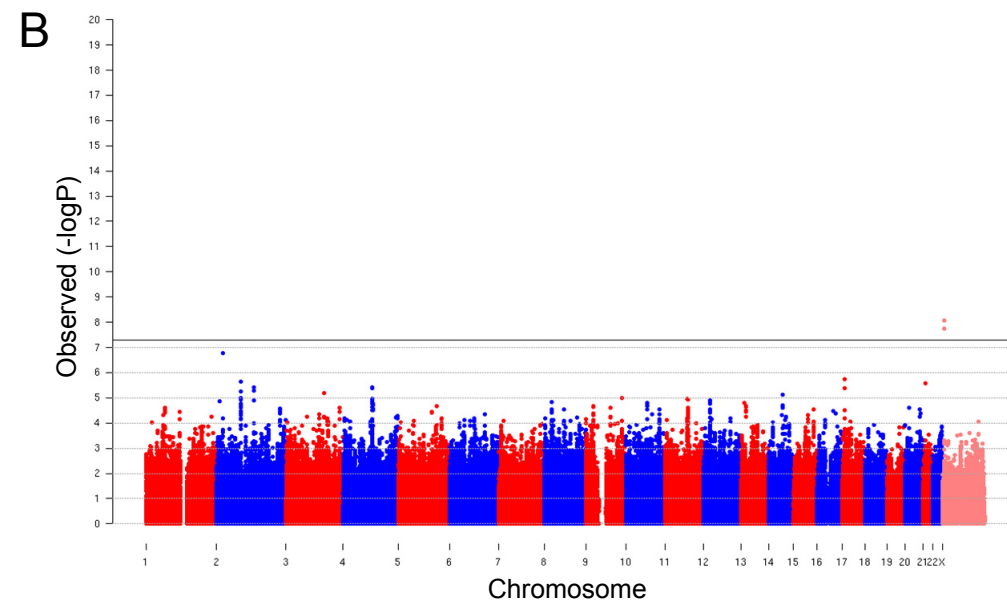

Supplement: Figure S1 — Manhattan plots giving genome-wide –log10 p-value according to chromosomal location for inverse-variance weighted meta-analysis of untransformed serum testosterone (A) and SHBG-adjusted serum testosterone (B) using an imputation quality filter (observed/expected variance ratio) >0.4 at the individual cohort level during meta-analysis. All seven discovery cohorts (n = 8,938) were included in the GWAS of chromosomes 1–22 while only the two largest cohorts (FHS and SHIP, n = 5,067) had GWAS data available for the X chromosome. (PDF) [file pgen.1002313.s001.pdf]

**Figure S2**

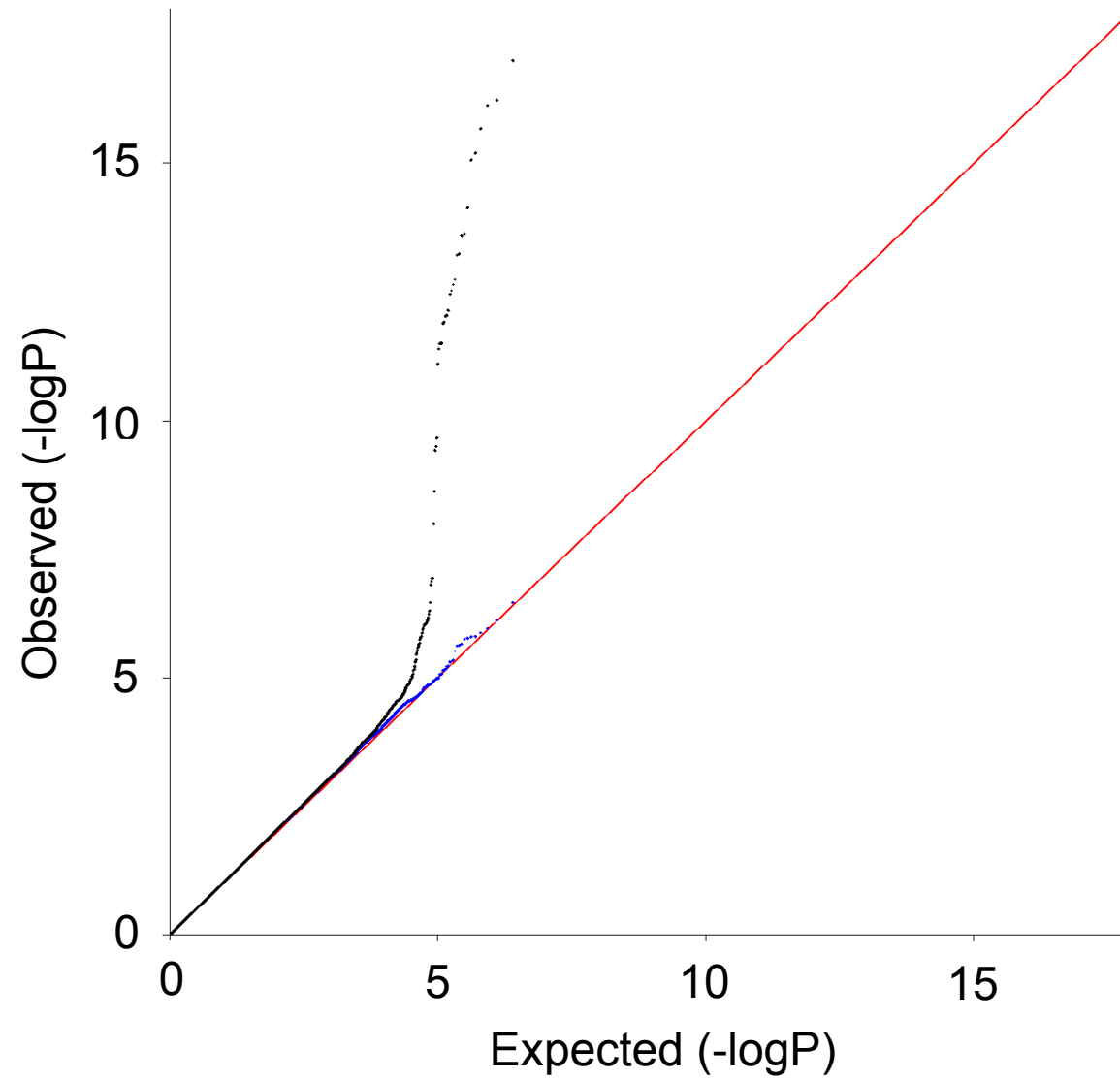

Supplement: Figure S2 — Quantile-quantile plot of the genome-wide association results of the inverse-variance weighted meta-analysis of untransformed serum testosterone including all SNPs (black) and after removal of the SNPs of the SHBG locus (blue). (PDF) [file pgen.1002313.s002.pdf]

**Figure S3**

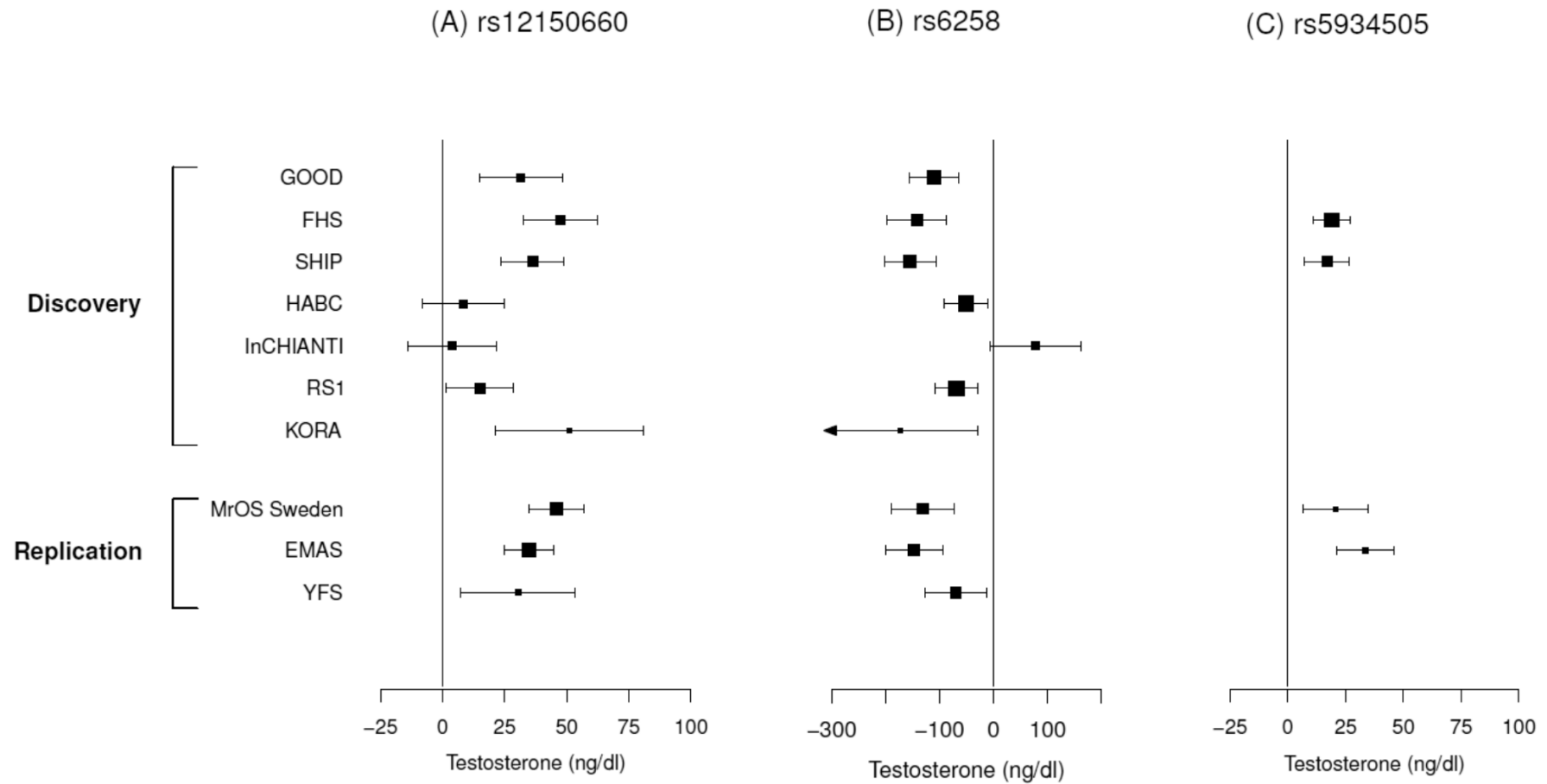

Supplement: Figure S3 — Associations for (A) rs12150660 and (B) rs6258 with testosterone and for (C) rs5934505 with SHBG-adjusted testosterone. Effects sizes are given per minor allele. Beta estimates and their 95% confidence intervals are given. The size of the data markers is proportional to the weight (inverse of the variance) of each study. (PDF) [file pgen.1002313.s003.pdf]

Figure S4

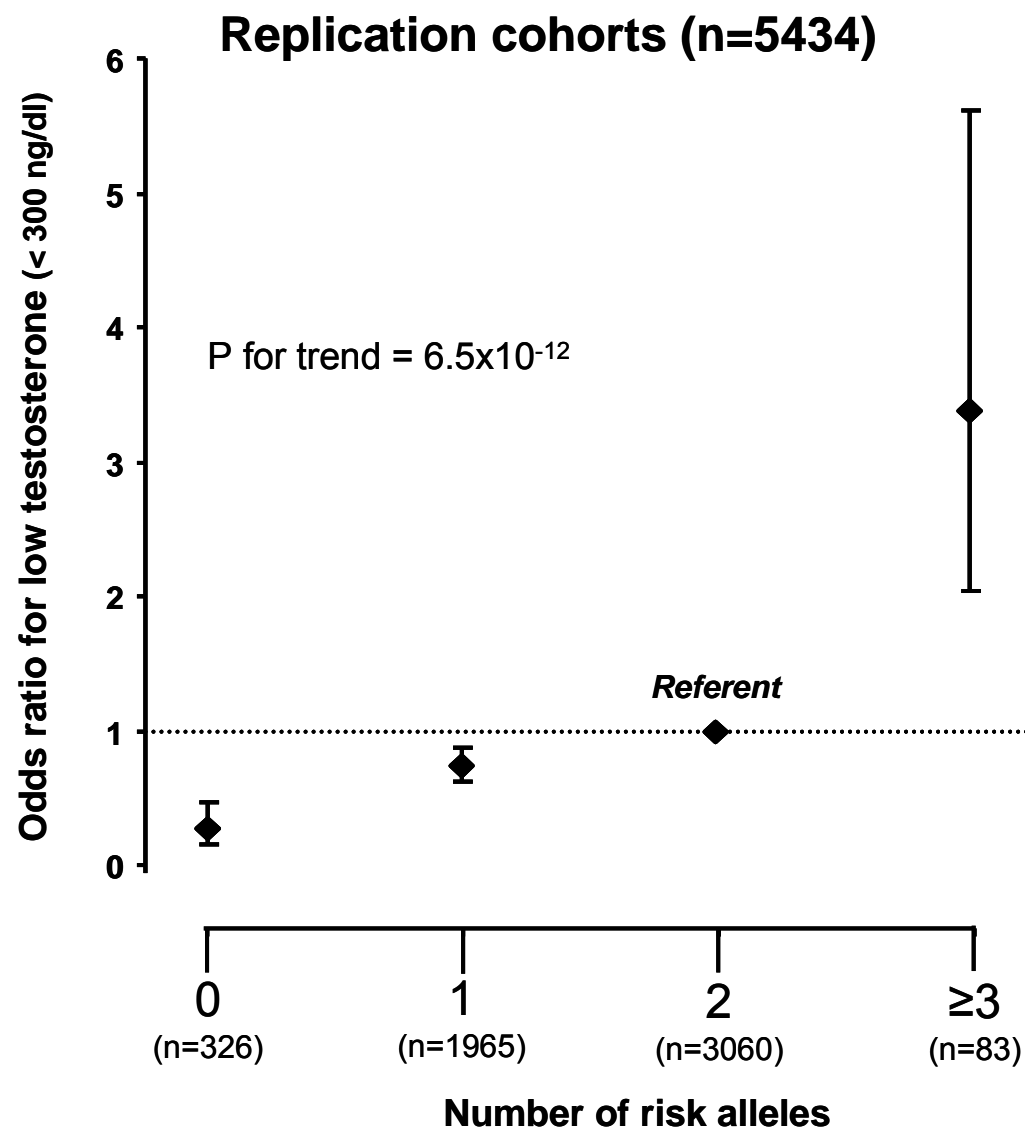

Supplement: Figure S4 — Risk of low serum testosterone concentrations (serum testosterone <300 ng/dl), according to the number of combined risk alleles for rs12150660 (G = risk allele) and rs6258 (T = risk allele) in the three replication cohorts (MrOS Sweden, EMAS, and YFS). Bars indicate 95% confidence intervals. Only two individuals in the three replication cohorts had four risk alleles and therefore individuals with three and four risk alleles were pooled into one group with ≥3 risk alleles. Two risk allele counts were used as reference, since this is the most prevalent amount among the cohorts. (PDF) [file pgen.1002313.s004.pdf]

**Figure S5**

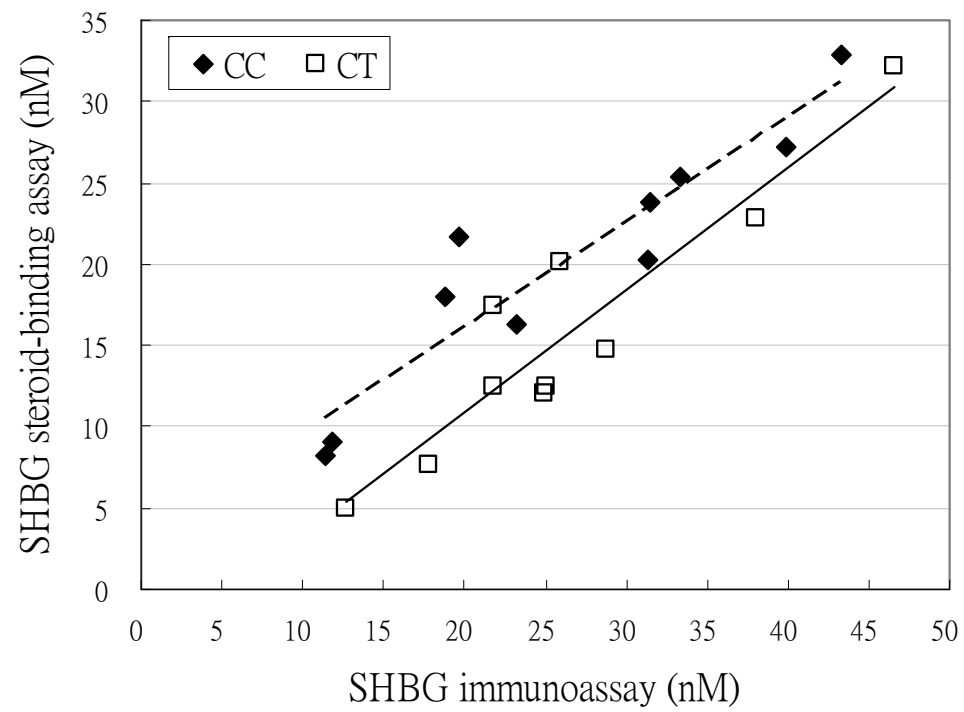

Supplement: Figure S5 — Subjects heterozygous for the SHBG allele containing an rs6258 SNP have lower serum SHBG steroid-binding capacity (Y-axis) when compared to the concentrations of SHBG measured by immunoassay (X-axis). Serum SHBG concentrations from 10 individuals homozygous for the wild type SHBG allele (CC, dashed regression line r2 = 0.872) or heterozygous for the rs6258 variant SHBG allele (CT, solid regression line r2 = 0.866) were measured by a time-resolved immunofluorometric assay[33], and a steroid-binding capacity assay using [3H]DHT as the labelled ligand.[34] (PDF) [file pgen.1002313.s005.pdf]
