# Supplementary material for: Genetic Determinants of Serum Testosterone Concentrations in Men
Source: PLoS Genet. 2011 Oct 6;7(10):e1002313. doi: 10.1371/journal.pgen.1002313 (PMC3188559; doi:10.1371/journal.pgen.1002313)
Supplement: Table S1 — Characteristics of 14,429 men from 10 cohorts included in the genome-wide association study meta-analysis. (PDF) [file pgen.1002313.s006.pdf]

Table S1

Supplemental Table 1: Characteristics of 14,429 men from 10 cohorts included in the genome-wide association study meta-analysis.

	FHS	SHIP	GOOD	KORA	HEALTH ABC	RS1	InChianti	EMAS	MrOS Sweden	YFS
	Discovery cohorts							Replication cohorts		
**N**	3151	1920	930	855	858	689	535	2340	2280	871
**Age, years**	49.2 (13.7)	51.4 (16.5)	18.9 (0.6)	61.07 (8.9)	73.9 (2.9)	68.5 (7.7)	67.1 (15.4)	59.7 (11.0)	75.4 (3.2)	31.7 (5.0)
**Serum testosterone, ng/dl**	588.8 (455.9; 761.0)	457.9 (362.9; 576.0)	458.0 (371.0; 552.3)	850.2* (660; 1022)	373 (212.5; 533.5)	334.9 (278.4; 399.9)	440.0 (366.0; 539.0)	455.0 (352.0; 570.0)	438.0 (340.0; 552.0)	541.5(335.4; 631.1)
**SHBG, nmol/l**	43.5 (30.6; 60.0)	30.3 (22.9; 39.6)	19.8 (15.2; 24.6)	27.7 (21.1; 35.6)	50.6 (21.5; 79.6)	34.2 (26.5; 42.8)	87.3 (62.7; 120.1)	38.9 (28.9; 51.7)	39.4 (29.2; 53.2)	29.4 (22.4; 36.7)
**Free testosterone, ng/dl**	10.1 (7.7, 13.4)	10.2 (8.1; 12.8)	12.9 (10.4; 15.7)	20.3 (16.5; 25.6)	5.0 (3.1; 8.8)	6.3 (4.8; 8.0)	4.4 (3.2; 6.1)	8.3 (6.8; 10.0)	7.9 (6.3; 9.9)	13.7 (11.5; 16.5)
**Current smoking, %**	14.9	33.7	8.6	16.7	5.5	30.8	25.6	22.0	8.5	30.0
**BMI, kg/m²**	28.3 (4.6)	27.6 (4.0)	22.3 (3.2)	28.3 (4.8)	27.1 (3.7)	25.7 (3.0)	27 (3.4)	27.8 (4.1)	26.3 (3.6)	25.8 (4.2)
**Serum sample ***	fasting	non-fasting	non-fasting	fasting	fasting	non-fasting	fasting	fasting	non-fasting	fasting
**Assay**	LC-MS/MS	CLIA	GC-MS	CLIA	CLIA	RIA	RIA	GC-MS	GC-MS	RIA

Continuous parameters are given as mean (SD) or median (Q1, Q3). To convert the values of serum testosterone to nmol/l divide by 28.82.

SHBG, sex hormone-binding globulin; BMI, body mass index; CLIA, chemiluminescent immunoassay; RIA, radioimmunoassay; GC-MS, gas chromatography-mass spectroscopy; LC-MS/MS, liquid chromatography tandem mass spectrometry.

* As testosterone concentrations in KORA were measured in plasma samples, we also performed the analyses after excluding the KORA data. Exclusion of the KORA cohort did not affect the overall results.
