# Supplementary material for: Genetic Determinants of Serum Testosterone Concentrations in Men
Source: PLoS Genet. 2011 Oct 6;7(10):e1002313. doi: 10.1371/journal.pgen.1002313 (PMC3188559; doi:10.1371/journal.pgen.1002313)
Supplement: Table S2 — Additional genotyping information for the 10 cohorts included in the genome-wide association study meta-analysis. (PDF) [file pgen.1002313.s007.pdf]

Table S2

Supplemental Table 2: Additional genotyping information for the 10 cohorts included in the genome-wide association study meta-analysis.

	FHS	SHIP	GOOD	KORA	HABC	RS1	InChianti	YFS	EMAS	MrOS Sweden
	Discovery cohorts							Replication cohorts		
**Genotyping platform & SNP panel**	Affymetrix 500K & 50K Human Gene Focused Panel	Affymetrix 6.0	Illumina HumanHap 610K	Affymetrix 6.0	Illumina Human1M-Duo	Illumina 550K bead array	Illumina 550K bead array	Illumina BeadChip Human670K	KASPar/Taqman	KASPar/Taqman
**Genotyping calling algorithm**	BRLMM	Birdseed2	BeadStudio	Birdseed	Illumina BeadStudio	Beadstudio Genecall	BeadStudio	Illuminus	NA	NA
**Average call rate**	97%	>98%	>97.5%	>98%	98%	97%	98%	=95%	97%	>98%
**Imputation software**	MACH v1.0.15	IMPUTE v0.5.0	MACH v1.0	IMPUTE v0.4.2	MACH v1.0.16	MACH V1.0.15	MACH v1.0	MACH v1.0	NA	NA
**Minor allele frequency**										
**rs6258** (HapMap-CEU: 1.8%)	1.8%	1.8%	2.2%	1.2%	3.0%	1.7%	0.9%	2.9%	1.5%	1.4%
**rs12150660** (HapMap-CEU: 20.0%)	23.0%	24.5%	21.7%	25.3%	22.7%	23.6%	23.8%	23.9%	24.8%	23.9%
**rs5934505** (HapMap-CEU: 24.8%)	27.9%	23.5%	NA	NA	NA	NA	NA	NA	26.8%	23.1%
**Imputation quality**										
**rs6258**	0.64	0.68	0.97	0.58	0.98	0.90	0.93	1.00	genotyped	genotyped
**rs12150660**	0.81	0.94	0.95	0.90	0.99	0.95	0.95	0.97	genotyped	genotyped
**rs5934505**	0.94	0.97	NA	NA	NA	NA	NA	NA	genotyped	genotyped
