# Supplementary material for: Genetic Determinants of Serum Testosterone Concentrations in Men
Source: PLoS Genet. 2011 Oct 6;7(10):e1002313. doi: 10.1371/journal.pgen.1002313 (PMC3188559; doi:10.1371/journal.pgen.1002313)
Supplement: Table S3 — Meta Analysis of untransformed total testosterone using Random Effect Model. (PDF) [file pgen.1002313.s008.pdf]

Table S3

Supplemental Table 3: Meta Analysis of untransformed total testosterone using Random Effect Model					
All cohorts					
	**beta**	**se**	**pval**	**tau²**	**H²**
**rs12150660**					
**Discovery**	26,73	6,78	7,99E-05	242.9266 (SE = 184.5716)	4,58
**Replication**	38,67	4,71	2,12E-16	22.8676 (SE = 67.3500)	1,51
**Combined**	30,19	5,05	2,29E-09	184.6676 (SE = 119.0002)	4,28
**rs6258**					
**Discovery**	-72,63	29,29	1,32E-02	4900.7863 (SE = 3433.1866)	7,97
**Replication**	-102,25	23,74	1,65E-05	887.9061 (SE = 1691.2813)	2,11
**Combined**	-81,84	20,49	6,51E-05	3235.9179 (SE = 1952.9804)	5,48
Without InCHIANTI					
	**beta**	**se**	**pval**	**tau²**	**H²**
**rs12150660**					
**Discovery**	30,33	6,73	6,53E-06	195.2204 (SE = 170.5274)	3,96
**Replication**	38,67	4,71	2,12E-16	22.8676 (SE = 67.3500)	1,51
**Combined**	32,92	4,69	2,22E-12	131.0489 (SE = 97.3849)	3,41
**rs6258**					
**Discovery**	-92,68	19,53	2,09E-06	1485.2949 (SE = 1408.4870)	3,35
**Replication**	-102,25	23,74	1,65E-05	887.9061 (SE = 1691.2813)	2,11
**Combined**	-95,19	14,60	6,95E-11	1136.7044 (SE = 933.6493)	2,68
