# Supplementary material for: Genetic Determinants of Serum Testosterone Concentrations in Men
Source: PLoS Genet. 2011 Oct 6;7(10):e1002313. doi: 10.1371/journal.pgen.1002313 (PMC3188559; doi:10.1371/journal.pgen.1002313)
Supplement: Text S1 — Supplemental methods. (DOC) [file pgen.1002313.s009.doc]

**Text S1**

**Statistical analyses**

To find single-nucleotide polymorphisms (SNPs) that are independently associated with the outcome within the same region, SNPs were “clumped together” using the clumping algorithm of PLINK (r2 > 0.1, 1 Mb distance) based on genotypes from HapMap release 27 CEU dataset. To verify the two independent associations of rs12150660 and rs6258 found, both SNPs together were used as additional covariates in the corresponding multivariate regression models. In the discovery stage inverse-variance meta-analysis of SHBG adjusted testosterone concentrations, some SNPs reached genome-wide significance which disappeared after applying an imputation quality filter (observed/expected variance ratio) > 0.4 at the individual cohort level during meta-analysis. Therefore, associations with these SNPs were considered as spurious results due to low imputation quality and were not taken into the replication stage. For all other analyses, no imputation quality filter was applied. For quality control purposes, the association p-values of each model of the meta-analysis were correlated pairwise but separately for SHBG adjusted and unadjusted analyses. The results were highly correlated (pairwise r² ≥ 0.8, MAF > 1% filter). The associations of rs12150660 and rs6258, respectively, with SHBG (nmol/L) and calculated free testosterone concentrations (ng/dl) were evaluated in a similar manner as for serum total testosterone concentrations (adjusted for age, BMI, and current smoking).

**Free testosterone fraction**

Free testosterone fraction was measured by an equilibrium dialysis method in 87 subjects with the CC genotype and 32 subjects with the CT genotype of rs6258 (Figure 3D) . Dialysis cells (The Nest Group Inc Southborough, MA) were used to separate the dialyzable (free) and bound fractions of testosterone. One hundred microliters of serum, to which ^3^H-labeled testosterone had been added, was dialyzed against 2.4 mL dialysis buffer, designed to approximate the composition of a protein-free ultrafiltrate of normal human serum. We have shown previously that equilibrium is reached in dialysis in 8 hours; no significant differences in the dialyzed fraction of testosterone were detected after 8, 16, and 24 hours of dialysis. Therefore, dialysis was performed overnight for 16 hours at 37°C . The dialysate from the outer compartment was counted in a scintillation counter to determine the fraction of ^3^H-testosterone that was dialyzed.

**Study specific cohort information**

***Framingham Heart Study (FHS)***

The FHS is a National Heart, Lung and Blood Institute contract-funded longitudinal cohort study initiated in 1948 to study determinants of cardiovascular disease and other major illnesses. The Original Cohort comprised 5,209 men and women, aged 28-62 years at enrollment . In 1971, 5,124 Offspring of the Original Cohort participants and Offspring spouses, aged 5 to 70 years, were enrolled into the Framingham Offspring Study. Offspring participants have been examined approximately every 4 to 8 years . In the 1990s, DNA was obtained for genetic studies from surviving Original cohort and Offspring participants. From 2002 to 2005, 4095 children of the Offspring participants including 1,912 men, mean age 40 years (range 19 to 72 years) were enrolled into the Framingham Third Generation cohort (Gen 3) and underwent a routine examination that included a blood sample for genetic studies . Sex hormones were measured at offspring exam 7 (1998-2001) and Gen 3 exam 1. Among the 3,537 men attending the exams, men with missing testosterone measurement (n=171) or genotype or covariate data (n= 207) and men with prostate cancer undergoing androgen deprivation therapy (n=8) were excluded resulting in a sample of 3,151 men. FHS examinations were approved by the Institutional Review Board of the Boston University Medical Center and all participants provided written informed consent.

*Genotyping information*

Genotyping was conducted through the FHS SHARe (SNP Health Association Resource) project initiated in 2007 on all Original Cohort, Offspring, and Gen 3 participants with DNA using the Affymetrix 500K mapping array in addition to the Affymetrix 50K supplemental array (http://www.ncbi.nlm.nih.gov/projects/gap/cgi-bin/study.cgi?study_id=phs000007). Sample level exclusions included a participant call rate <97%, a per subject heterozygosity ±5 standard deviations away from the mean, or a per subject large Mendelian error rate. Principal components analysis was used to infer axes of variation using a subset of 425,173 SNPs with MAF ≥0.01, HWE p ≥10-8, and call rate ≥0.95. SNP weights for 10 principal components (PCs) were inferred using a maximal set of independent individuals. PCs 1 and 8 were significantly associated with testosterone (p<.01), and were included as covariates in the SNP association analyses. MACH (version 1.0.15) was used to impute all autosomal SNPs on HapMap, using the publicly available phased haplotypes from HapMap (release 22, build 36, CEU population) as a reference panel . From a total of 534,982 genotyped autosomal SNPs (Affymetrix 500K and 50K combined), we used 378,163 SNPs that were present on HapMap and that passed quality control measures in the FHS sample including MAF ≥0.01, HWE p>10-6, callrate >0.97, mishap test of non-random missingness p>10-9, and ≤100 Mendelian errors. IMPUTE v0.5 was used to impute X chromosome SNPs. From a total number of 10,886 Chromosome X genotyped SNPs, 7,795 SNPs were used as input to the IMPUTE program after the removal of SNPs that did not meet our quality control measures or were not included in the HapMap legend files.

*Serum Testosterone assays*

Serum testosterone, free testosterone and sex hormone binding globulin (SHBG) were measured in the morning after an overnight fast of about 10 hours, typically between 7.30 AM and 8.30 AM in men attending Offspring examination 7 (1998-2001) and Gen 3 examination 1 (2002-2005). The samples were aliquoted and immediately stored frozen at -80C and remained frozen until the time of assay. We measured serum testosterone by liquid chromatography tandem mass spectrometry using a NIST standard, as described . As part of the Centers for Disease Control’s (CDC) Testosterone Assay Harmonization Initiative, 40 quality control samples were run after each batch of 200 FHS samples. In addition, 28 serum samples from men and women with testosterone concentrations across the entire male and female range were measured in a blinded manner in the Boston University and Mayo laboratories. The Pearson correlation between the values obtained in the two laboratories was greater than 0.99 and Bland-Altman plots revealed no significant differences between values obtained in the two laboratories at any concentration. The functional sensitivity of the assay was 2 ng/dL, and inter-assay CV 7.8%, 5.9%, and 3.5%, respectively, in samples with testosterone concentrations of 250, 500, and 1000 ng/dL. Serum SHBG concentrations were measured using a DELFIA™ immunofluorometric assay (PerkinElmer Life Sciences, Turku, Finland). The intra-assay CVs were 8.3%, 7.9%, and 10.9% in the low, medium, and high pools . Free testosterone was for all participating cohorts calculated by using a modified law of mass action equation, as described by Mazer .

*Acknowledgments*

The FHS phenotype-genotype analyses for this work were supported by the National Institute of Aging (Genetics of Reproductive Life Period and Health Outcomes, R21AG032598; JMM, KL, DK, DPK, WVZ). The Framingham Heart Study of the National Heart Lung and Blood Institute of the National Institutes of Health and Boston University School of Medicine is supported by the National Heart, Lung and Blood Institute's Framingham Heart Study Contract No. N01-HC-25195 and its contract with Affymetrix, Inc for genotyping services (Contract No. N02-HL-6-4278). Sex hormone measurements were funded primarily by National Institute on Aging grant- 1RO1AG31206(PIs: SB and VSR); additional support was provided by the Boston Claude D. Pepper Older Americans Independence Center (5P30AG031679) and a grant from the National Institute on Aging and the National Institute of Arthritis Musculoskeletal and Skin Diseases to Dr. Kiel [DPK] (R01 AR/AG 41398). Analyses reflect intellectual input and resource development from the Framingham Heart Study investigators participating in the SNP Health Association Resource (SHARe) project. A portion of this research was conducted using the Linux Cluster for Genetic Analysis (LinGA-II) funded by the Robert Dawson Evans Endowment of the Department of Medicine at Boston University School of Medicine and Boston Medical Center.

*Disclosures*

Dr. Bhasin has received research grants from Solvay Pharmaceuticals, Merck and Co., and Ligand Pharmaceuticals for research unrelated to the contents of this manuscript. He serves as a member of the American Board of Internal Medicine, Endocrinology Metabolism and he chaired the Endocrine Society’s Expert Panel for the development of guideline for Testosterone Therapy of Androgen Deficiency Syndromes in Men.

***Study of Health in Pomerania (SHIP)***

SHIP is a longitudinal cohort study in West Pomerania, the north-east area of Germany . From the entire study population of 212,157 inhabitants living in the area, a sample was selected from the population registration offices, where all German inhabitants are registered. Only individuals with German citizenship and main residency in the study area were included. A two-stage cluster sampling method was adopted from the WHO MONICA Project Augsburg, Germany. In a first step, the three cities of the region (with 17,076 to 65,977 inhabitants) and the 12 towns (with 1,516 to 3,044 inhabitants) were selected. Further 17 out of 97 smaller towns (with less than 1,500 inhabitants) were drawn at random. In a second step, from each of the selected communities, subjects were drawn at random, proportional to the population size of each community and stratified by age and gender. Finally, 7,008 subjects aged 20 to 79 years were sampled, with 292 persons of each gender in each of the twelve five-year age strata. In order to minimize drop-outs by migration or death, subjects were selected in two waves. The net sample (without migrated or deceased persons) comprised 6,267 eligible subjects. Selected persons received a maximum of three written invitations. In case of non-response, letters were followed by a phone call or by home visits if contact by phone was not possible. The SHIP population finally comprised 4,308 participants at baseline (corresponding to a final response of 68.8%). Of the 2,116 male participants, men with missing genotype or phenotype data were excluded, as well as participants reporting chemical or surgical castration or intake of sexual hormones (anatomic-therapeutical-chemical [ATC] code G03), testosterone 5α reductase inhibitors (G04CB), or sexual hormone antagonists (L02B). Altogether, valid data were available in 2,027 males of the SHIP baseline.

*Genotyping information*

The SHIP samples were genotyped using the Affymetrix Human SNP Array 6.0. Hybridisation of genomic DNA was done in accordance with the manufacturer’s standard recommendations. The genetic data analysis workflow was created using the Software InforSense. Genetic data were stored using the database Caché (InterSystems). Genotypes were determined using the Birdseed2 clustering algorithm. For quality control purposes, several control samples where added. On the chip level, only subjects with a genotyping rate on QC probesets (QC callrate) of at least 86% were included. All remaining arrays had a sample callrate >92%. The overall genotyping efficiency of the GWA was 98.55 %. Imputation of genotypes in SHIP was performed with the software IMPUTE v0.5.0 based on HapMap II. Genome-wide association tests were performed using QUICKTEST v0.95 (http://toby.freeshell.org/software/quicktest.shtml). Uncertainties for imputed genotypes were taken into account for association testing.

*Serum Testosterone assays*

Serum testosterone concentrations were measured from frozen serum aliquots using competitive chemiluminescent enzyme immunoassays on an Immulite 2500 analyzer (Siemens Immulite 2500, ref. L5KTW, lot 110; Siemens Healthcare Medical Diagnostics, Bad Nauheim, Germany). Measurement was carried out from December 2005 to January 2006. An aliquot of two alternating levels of a third party commercial control material (Bio-Rad Lyphochek Immunoassay Plus Control, lot 40151 and lot 40152; Bio-Rad, Munich, Germany) was included in each series in single determination. The inter-assay coefficient of variation was 13.2% with a systematic deviation of +2.3 % at the 92.2 ng/dl level, and 8.9% with a systematic deviation of +0.24 % at the 648.5 ng/dl level .

*Acknowledgments*

Computing resources have been made available by the Leibniz Supercomputing Centre of the Bavarian Academy of Sciences and Humanities (HLRB project h1231). SHIP is part of the Community Medicine Research net of the University of Greifswald, Germany, which is funded by the Federal Ministry of Education and Research (grants no. 01ZZ9603, 01ZZ0103, and 01ZZ0403), the Ministry of Cultural Affairs as well as the Social Ministry of the Federal State of Mecklenburg - West Pomerania. Genome- wide data have been supported by the Federal Ministry of Education and Research (grant no. 03ZIK012) and a joint grant from Siemens Healthcare, Erlangen, Germany and the Federal State of Mecklenburg West Pomerania. The University of Greifswald is a member of the 'Center of Knowledge Interchange' program of the Siemens AG. This work is also part of the research project Greifswald Approach to Individualized Medicine (GANI_MED). The GANI_MED consortium is funded by the Federal Ministry of Education and Research and the Ministry of Cultural Affairs of the Federal State of Mecklenburg – West Pomerania (03IS2061A). The testosterone reagents used were sponsored by Siemens Healthcare Diagnostics, Eschborn, formerly DPC Biermann GmbH, Bad Nauheim, Germany. Novo Nordisk provided partial grant support for the determination of serum samples and data analysis.

*Disclosures*

Dr. Wallaschofski has received research grants from Novo Nordisk and Pfizer for research unrelated to the contents of this manuscript and honorarium for lectures by Merck and Co.

***Gothenburg Osteoporosis and Obesity Determinants (GOOD) Study***

The GOOD study was initiated to determine both environmental and genetic factors involved in the regulation of bone and fat mass . Male study subjects were randomly identified in the greater Gothenburg area in Sweden using national population registers, contacted by telephone, and invited to participate. To be enrolled in the GOOD study, subjects had to be between 18 and 20 years of age. There were no other exclusion criteria, and 49% of the study candidates agreed to participate (n = 1,068). The study was approved by the ethics committee at the University of Gothenburg. Written and oral informed consent was obtained from all study participants.

*Genotyping information*

Genotyping was performed using the Illumina HumanHap610 Quad arrays at the Genetic Laboratory, Department of Internal Medicine, Erasmus Medical Center, Rotterdam, Netherlands. Genotypes were called using the BeadStudio calling algorithm. Genotypes from 938 individuals passed the sample quality control criteria [exclusion criteria: sample call rate < 97.5%, gender discrepancy with genetic data from X-linked markers, excess autosomal heterozygosity > 0.33 ~ FDR < 0.1%, duplicates and/or first degree relatives identified using IBS probabilities (> 97%), ethnic outliers (3 SD away from the population mean) using multi-dimensional scaling analysis with four principal components]. Across 22 duplicate samples, genotype concordance exceeded 99.9%. Genotypes were imputed for all polymorphic SNPs (521,160 with MAF ≥ 1%, SNP call rate ≥ 98% and HWE p value ≥ 10^-6^) using the MACH software, based upon phased autosomal chromosomes of the HapMap CEU Phase II panel (release 22, build 36), orientated on the positive strand. We carried out association testing for imputed SNPs using a linear regression framework under an additive (per allele) genetic model as implemented in MACH2QTL . Uncertainty in genotype prediction was accounted for by utilizing the dosage information from MACH.

*Serum Testosterone assays*

A validated gas chromatography/mass spectroscopy system was used for the analysis of serum testosterone on frozen serum aliquots (limit of detection, 5 ng/dl; intra-assay CV, 2.9%; interassay CV, 3.4%) . Serum SHBG was measured using IRMA (Orion Diagnostics, Espoo, Finland; limit of detection, 1.3 nM; intra-assay CV, 3%; interassay CV, 7%).

*Acknowledgments*

Financial support was received from the Swedish Research Council (K2010-54X-09894-19-3, 2006-3832 and K2010-52X-20229-05-3), the Swedish Foundation for Strategic Research, the ALF/LUA research grant in Gothenburg, the Lundberg Foundation, the Torsten and Ragnar Söderberg's Foundation, Petrus and Augusta Hedlunds Foundation, the Västra Götaland Foundation, the Göteborg Medical Society, the Novo Nordisk foundation, the Canadian Institutes of Health Research (MOP-15261), and the European Commission grant HEALTH-F2-2008-201865-GEFOS. We would like to acknowledge Maria Nethander at the genomics core facility at University of Gothenburg for statistical analyses. We would also like to thank Dr. Tobias A. Knoch, Luc V. de Zeeuw, Anis Abuseiris, and Rob de Graaf as well as their institutions the Erasmus Computing Grid, Rotterdam, The Netherlands, and especially the national German MediGRID and Services@MediGRID part of the German D-Grid, both funded by the German Bundesministerium fuer Forschung und Technology under grants #01 AK 803 A-H and # 01 IG 07015 G for access to their grid resources. We would also like to thank Karol Estrada, Department of Internal Medicine, Erasmus MC, Rotterdam, Netherlands for advice regarding the grid resources.

*Disclosures*

No disclosures.

***Cooperative Research in the Region of Augsburg (KORA)***

The KORA study is a series of independent population-based epidemiological surveys and follow-up studies of participants living in the region of Augsburg, Southern Germany. All participants are residents of German nationality identified through the registration and informed consent has been given by all participants . The studies have been approved by the local ethics committee. The present study includes data of the follow-up study KORA F4 (2006-2008) of the KORA S4 survey (1999/2000). For genotyping, we included 1,814 randomly selected participants of KORA F4. Of the male participants, men with missing genotype or phenotype data were excluded, as well as participants reporting chemical or surgical castration or intake of sexual hormones (anatomic-therapeutical-chemical [ATC] code G03), testosterone 5α reductase inhibitors (G04CB), or sexual hormone antagonists (L02B). Altogether, valid data were available in 858 males of the KORA F4 survey.

*Genotyping information*

The KORA F4 samples were genotyped with the Affymetrix Human SNP Array 6.0. Hybridisation of genomic DNA was done in accordance with the manufacturer’s standard recommendations. Genotypes were determined using Birdseed2 clustering algorithm. For quality control purposes, we applied a positive control and a negative control DNA every 96 samples. On chip level only subjects with overall genotyping efficiencies of at least 93% were included resulting in an average genotyping efficiency of 98% per chip. In addition the called sex had to agree with the sex in the KORA study database. Imputation of genotypes was performed with the software IMPUTE v0.4.2 based on HapMap II. Genome-wide association tests were performed using SNPTEST v1.1.5, SNPTEST v2.1.1 and QUICKTEST.

*Serum Testosterone assays*

In the KORA cohort, plasma testosterone concentrations were determined from fasting EDTA plasma samples stored at -80°C until analysis. An automated chemiluminescent immunometric assays was used (Siemens Immulite 2000 Total Testosterone, ref. L2KTW2, lot 214; Siemens Healthcare Medical Diagnostics, Bad Nauheim, Germany). The assays were performed according to the manufacturer’s recommendations by skilled technical personal in September and October 2009. Third party commercial control material at three different levels (Bio-Rad CON6 Multivalent Control Module, lot 022; Bio-Rad, Munich, Germany) was included in each series. The inter-assay coefficients of variation were 12.2%, 8.9% and 4.2% at serum testosterone concentrations of 117 ng/dl, 333 ng/dl and 1108 ng/dl, respectively. All samples and controls were assayed without replication.

*Acknowledgments*

The KORA research platform was initiated and financed by the Helmholtz Center Munich, German Research Center for Environmental Health, which is funded by the German Federal Ministry of Education and Research (BMBF) and by the State of Bavaria. Part of this work was financed by the German National Genome Research Network (NGFN-2 and NGFNPlus: 01GS0823). Our research was supported within the Munich Center of Health Sciences (MC Health) as part of LMUinnovativ. This study was in part supported by a grant from the German Federal Ministry of Education and Research (BMBF) to the German Center for Diabetes Research (DZD e.V.).

*Disclosures*

No disclosures.

***Health, Aging, and Body Composition (Health ABC) Study***

The Health ABC study is a longitudinal cohort study consisting of 3,075 initially well-functioning, community-dwelling, 70- to 79-year old, black and white men and women. Participants were identified from a random sample of white Medicare beneficiaries and all age-eligible black residents in designated zip code areas surrounding Memphis, Tennessee, and Pittsburgh, Pennsylvania. Participants were eligible if they reported no difficulty in walking one quarter of a mile, going up 10 steps without resting and performing basic activities of daily living. Participants were excluded if they reported a history of active treatment for cancer in the prior three years, planned to move out of the study area in the next three years, or were currently participating in a randomized trial of a lifestyle intervention. Baseline data, collected between April 1997 and June 1998, included an in-person interview and a clinic-based examination, with evaluation of body composition, clinical and sub-clinical diseases, and physical functioning. All participants signed informed written consent forms approved by the institutional review boards of the clinical sites. Of the 939 white men, men with missing genotype or phenotype data were excluded as well as men who took sexual hormones, testosterone, or sexual hormone antagonist. For the present analysis, 785 white men were included.

*Genotyping information*

Genomic DNA was extracted from buffy coat collected using PUREGENE® DNA Purification Kit during the baseline exam. In 2009, genotyping was performed by the Center for Inherited Disease Research (CIDR) using the Illumina Human1M-Duo BeadChip system. Samples were excluded from the dataset for the reasons of sample failure, genotypic sex mismatch, and first degree relative of an included individual based on genotype data. Genotyping was successful in 2,802 individuals (1663 Caucasians and 1139 African Americans). Genotypes were available on 914263 high quality SNPs before imputation.

*Serum Testosterone assays*

In the Health ABC study, serum was obtained after an overnight fast and kept frozen until determination of serum testosterone, was measured using IMMULITE®, an automated continuous random-access chemiluminescent immunoassay (CLIA) system (Diagnostic Products Corporation, Los Angeles, CA). Serum testosterone has a detectable range of 0.7–55.5 nmol/l. Each sample was run in duplicate and an additional 147 'blinded' samples (5%) were collected and included for measurement of each hormone assay to determine assay reproducibility and showed a CV of 10.8% for serum testosterone .

*Acknowledgments*

This study was supported by National Institute on Aging contracts N01-AG-6-2101, N01-AG-6-2103, and N01-AG-6-2106. The genome-wide association study was funded by NIA grant 1R01AG032098-01A1 to Wake Forest University Health Sciences and genotyping services were provided by the Center for Inherited Disease Research (CIDR). CIDR is fully funded through a federal contract from the National Institutes of Health to The Johns Hopkins University, contract number HHSN268200782096C.” This research was supported (in part) by the Intramural Research Program of the NIH, National Institute on Aging.

*Disclosures*

No disclosures.

***Rotterdam study (RS1)***

Subjects were participants of the RS1, a large prospective population-based cohort study of Caucasian subjects aged 55 years and over, living in the Ommoord district of Rotterdam, the Netherlands. The study was designed to investigate the incidence and determinants of chronic disabling diseases in the elderly. Rationale and design have been described previously . All 10,275 inhabitants aged 55 years and over were invited for baseline examination between August 1990 and June 1993. Of those, 7,983 participated. Among the subjects living independently, the overall response rate was 77 percent for home interview and 71 percent for examination in the research centre, where anthropometric characteristics and bone mineral density were measured, and blood samples were taken. The Rotterdam Study was approved by the medical ethics committee of the Erasmus University Medical School, and written informed consent was obtained from each subject. The current study is based on 640 men for whom genome-wide genotype data and serum testosterone concentrations were available.

*Genotyping information*

The Rotterdam Study samples were genotyped using the Illumina Infinium HumanHap550 Beadchip. The following sample QC criteria were applied in the GWAS of RS-I, sample call rate ≥97.5%, gender mismatch with typed X-linked markers, evidence for DNA contamination in the samples using the mean of the autosomal heterozygosity >0.33, exclusion of duplicates or first-degree relatives identified using IBS probabilities, exclusion of outliers (three SD away from the population mean) using multi-dimensional scaling (MDS) analysis with four PC. The exclusion/filtering criteria for SNPs were the following: Minor Allele Frequency ≥ 1%, SNP-callrate ≥ 97.5% and HWE-pvalue ≥ 10-6. Genotypes were imputed for all polymorphic SNPs (minor allele frequency >0.01) using the MACH software, based upon phased autosomal chromosomes of the HapMap CEU Phase II panel (release 22, build 36), orientated on the positive strand. Imputation QC metrics from MACH were used for filtering out SNPs with low-quality data. Statistical analysis of imputed data was performed using MACH2QTL implemented in GRIMP .

*Serum Testosterone assays*

Non-fasting blood samples were drawn by venipuncture at the baseline examination in the research center between 0830 and 1600 h. For the collection of serum, blood was sampled in 5-ml tubes containing 0.5-ml sodium citrate solution. Platelets were removed by centrifugation, and the samples were stored at –80 C until hormone measurements. The period of storage of frozen serum varied from 7.5–12.5 yr. Testosterone was estimated in single measurements by RIA using coated tubes (Diagnostic Systems Laboratories, Webster, TX). The interassay coefficient of variation, determined on basis of duplicate results of internal quality control serum pools with three different levels of analytes was 12%.

*Acknowledgments*

The generation and management of GWAS genotype data for the Rotterdam Study is supported by the Netherlands Organisation of Scientific Research NWO Investments (nr. 175.010.2005.011, 911-03-012). This study is funded by the Research Institute for Diseases in the Elderly (014-93-015; RIDE2), the Netherlands Genomics Initiative (NGI) - Netherlands Consortium of Healthy Aging (NCHA) project nr. 050-060-810, and funding from the European Commision (HEALTH-F2-2008-201865, GEFOS; HEALTH-F2-2008-35627, TREAT-OA). The Rotterdam Study is funded by Erasmus Medical Center and Erasmus University, Rotterdam, Netherlands Organization for the Health Research and Development (ZonMw), the Research Institute for Diseases in the Elderly (RIDE), the Ministry of Education, Culture and Science, the Ministry for Health, Welfare and Sports, the European Commission (DG XII), and the Municipality of Rotterdam. We thank Pascal Arp, Mila Jhamai, Dr Michael Moorhouse, Marijn Verkerk, and Sander Bervoets for their help in creating the GWAS database. The authors are grateful to the study participants, the staff from the Rotterdam Study and the participating general practioners and pharmacists. We would like to thank Dr. Tobias A. Knoch, Luc V. de Zeeuw, Anis Abuseiris, and Rob de Graaf as well as their institutions the Erasmus Computing Grid, Rotterdam, The Netherlands, and especially the national German MediGRID and Services@MediGRID part of the German D-Grid, both funded by the German Bundesministerium fuer Forschung und Technology under grants #01 AK 803 A-H and # 01 IG 07015 G for access to their grid resources.

*Disclosures*

No disclosures.

***Invecchiare in Chianti (InCHIANTI)***

The InCHIANTI study is a population-based epidemiological study aimed at evaluating factors that influence mobility in the older population living in the Chianti region of Tuscany, Italy. Details of the study have been previously reported . Briefly, 1616 residents were selected from the population registry of Greve in Chianti (a rural area; 11,709 residents with 19.3% of the population greater than 65 years of age) and Bagno a Ripoli (Antella village near Florence; 4,704 inhabitants, with 20.3% greater than 65 years of age). The participation rate was 90% (n= 1,453) and participants ranged between 21–102 years of age. The study protocol was approved by the Italian National Institute of Research and Care of Aging Institutional Review. There were 85 parent-offspring pairs, 6 sib-pairs and 2 half-sibling pairs documented. We investigated any further familial relationships using IBD of 10,000 random SNPs using RELPAIR and uncovered 1 parent-offspring, 79 siblings and 13 half-sibling . We utilized the correct family structure inferred from genetic data for all analyses. There were 535 males with GWAS data and serum testosterone measured.

*Genotyping information*

Genome wide genotyping was performed using the Illumina Infinium HumanHap550 genotyping chip (ver1 and ver3 chips were used), using 750ng of genomic DNA extracted from whole blood. Chips were scanned on Illumina BeadStation scanners and data analyzed in BeadStudio (version 3; Illumina). We only used DNA samples for which >98% of all SNPs were scored, that were called in >98% of samples and had minor allele frequencies in our sample of >1%. SNPs deviating appreciably from the expected population distribution (Hardy Weinberg Equilibrium p<1x10^-4^) were also excluded from the analyses. We imputed 2.5 million HapMap SNPs using IMPUTE (http://www.stats.ox.ac.uk/~marchini/software/gwas/impute). Imputation quality was determined by calculated empirical variance divided by the expected variance (oevar) and for the ‘proper info’ output variable was used to determine imputation quality. Analysis was performed using linear regression allele dosage in SNPTEST (http://www.stats.ox.ac.uk/~marchini/software/gwas/snptest). We corrected for any over inflation of statistics due to relatedness or residual population admixture by using an inflation factor, generated using EIGENSTRAT.

*Serum Testosterone assays*

Serum testosterone concentrations were assessed through a commercial radioimmunological kit (Active Testosterone RIA DSL-4000; Diagnostic Systems Laboratories, Webster, TX, USA, distributed by Chematil, s.r.l., Angri (SA), Italy). The minimum detection limit was 0.08 ng/ml. Intra-assay CVs for three different concentrations ranged from 7.8% to 9.6%, whilst inter-assay CVs ranged from 8.4% to 9.1%.

*Acknowledgments*

The InCHIANTI study baseline (1998-2000) was supported as a “targeted project” (ICS110.1/RF97.71) by the Italian Ministry of Health and in part by the U.S. National Institute on Aging (Contracts: 263 MD 9164 and 263 MD 821336); the InCHIANTI Follow-up 1 (2001-2003) was funded by the U.S. National Institute on Aging (Contracts: N.1-AG-1-1 and N.1-AG-1-2111); the InCHIANTI Follow-ups 2 and 3 studies (2004-2010) were financed by the U.S. National Institute on Aging (Contract: N01-AG-5-0002); supported in part by the Intramural research program of the National Institute on Aging, National Institutes of Health, Baltimore, Maryland. JRBP is funded by a Sir Henry Wellcome Postdoctoral Research Fellowship (092447/Z/10/Z)

*Disclosures*

No disclosures.

***European Male Ageing Study (EMAS)***

The EMAS is a prospective, population-based study of ageing in middle aged and elderly European men. Men were recruited from population based sampling frames in seven centres: Florence (Italy), Leuven (Belgium), Lodz (Poland), Manchester (UK) Santiago del Compostella (Spain), Szeged (Hungary), Tartu (Estonia). Details regarding recruitment, response rates and assessments have been described previously . Participating centres were selected to provide geographical and socioeconomic diversity within Europe, and facilities to perform epidemiological surveys. Stratified random sampling was performed in each centre with the aim of recruiting one hundred men in each of four 10-year age bands: 40-49 years, 50-59 years, 60-69 years, and 70-79 years. Subjects were invited by letter to complete a postal questionnaire, and attend for an interviewer-assisted questionnaire and give a fasting blood sample. Subjects were recontacted usually within 4 weeks if they did not reply following a first letter. Ethical approval for the study was obtained in accordance with local institutional requirements in each centre. The self completed and interviewer-assisted standardized validated questionnaires (in local languages) gathered information on medical conditions, medications, and current smoking. Height and weight were measured in the standing position. BMI was calculated as body weight (kg) divided by the square of height (m). From a total of 2,960 participants, men with missing genotype or phenotype data were excluded. In addition, men with prevalent pituitary or testicular diseases or those who currently use medications which could affect pituitary/testicular functions (testosterone, dehydroepiandrosterone, anti-androgens, gondotropin releasing hormone agonists, glucocorticoids and psycholeptic agents), or sex steroids clearance (e.g. anti-convulsants) were excluded, giving 2,295 men in the analysis sample.

*Genotyping information*

Genomic DNA was extracted from venous blood samples using phenol-chloroform. All SNPs were genotyped by K-Biosciences ([www.kbioscience.co.uk](http://www.kbioscience.co.uk/)) using either a competitive allele specific PCR (KASPar) or Taqman^TM^ assay. SNP call rate was ≥97% and all SNPs were in Hardy-Weinberg equilibrium (p>0.05). Subjects with suspected non-European ancestry, due to the reporting of a parent or grandparent being born outside Europe or North America, were excluded from the analysis.

*Serum Testosterone assays*

A single fasting morning (before 10 a.m.) venous blood sample was obtained from all subjects. Serum was separated immediately after phlebotomy and stored at -80°C until assay at the end of the baseline study. A validated gas chromatography/mass spectroscopy (GC-MS) system was used for the analysis of serum testosterone (limit of detection, 5 ng/dl; intra-assay CV, 2.9%; interassay CV, 3.4%) on frozen serum aliquots . SHBG was measured by the Modular E170 platform electrochemiluminescence immunoassay (Roche Diagnostics, Mannheim, Germany).

*Acknowledgments*

The EMAS is funded by the Commission of the European Communities Fifth Framework Programme “Quality of Life and Management of Living Resources” Grant QLK6-CT-2001-00258 and supported by funding from the UK Arthritis Research Campaign. The EMAS Principal Investigator is Professor Frederick Wu, MD; Dept of Endocrinology, Manchester Royal Infirmary, UK. The “EMAS Study Group" consists of the following people: Gyorgy Bartfai, Steven Boonen, Felipe Casanueva, Joseph D Finn, Gianni Forti, Aleksander Giwercman, Thang S Han, Kate L Holliday, Ilpo T Huhtaniemi, Krzysztof Kula, Michael EJ Lean, David M Lee, Terence W O'Neill, Neil Pendleton, Margus Punab, Stephen R Pye, Alan J Silman, Abdelouahid Tajar, Wendy Thomson, Dirk Vanderschueren, and Frederick CW Wu. The authors wish to thank the men who participated in the eight countries and the research/nursing staff in the eight centres: C Pott, Manchester, E Wouters, Leuven, M Nilsson, Malmö, M del Mar Fernandez, Santiago de Compostela, M Jedrzejowska, Lodz, H-M Tabo, Tartu, A Heredi, Szeged for their data collection and C Moseley, Manchester for data entry and project coordination. Dr Vanderschueren is a senior clinical investigator supported by the Clinical Research Fund of the University Hospitals Leuven, Belgium. Dr. Boonen is a senior clinical investigator of the Fund for Scientific Research-Flanders, Belgium (F.W.O.-Vlaanderen). Dr. Boonen is holder of the Leuven University Chair in Metabolic Bone Diseases.

*Disclosures*

No disclosures.

***The Osteoporotic Fractures in Men Study – Sweden (MrOS Sweden)***

The MrOS study is a multicenter, prospective study including 3,014 elderly men in Sweden, Hong Kong (≅2,000), and the United States (≅6,000). The MrOS Sweden cohort consist of three sub-cohorts from three different Swedish cities (n=1,005 in Malmö, n=1,010 in Göteborg, and n=999 in Uppsala). Study subjects (men aged 69–80 years) were randomly identified using national population registers, contacted and asked to participate. To be eligible for the study, the subjects had to be able to walk without assistance, provide self-reported data, and sign an informed consent; there were no other exclusion criteria. The study was approved by the ethics committees at the Universities of Gothenburg, Lund, and Uppsala. Informed consent was obtained from all study participants .

*Genotyping information*

Genotyping of all SNPs was carried out on the entire MrOS Sweden cohort for whom DNA was available (n=2,280) by KBioscience ([http://www.kbioscience.co.uk](http://www.kbioscience.co.uk/)), who employ a novel form of competitive allele specific PCR (KASPar) and Taqman^TM^ system for genotyping. SNP call rate was ≥98% and all SNPs were in HWE.

*Serum Testosterone assays*

A validated gas chromatography/mass spectroscopy system was used for the analysis of serum testosterone (limit of detection, 5 ng/dl; intra-assay CV, 2.9%; interassay CV, 3.4%) on frozen serum aliquots . Serum SHBG was measured using IRMA (Orion Diagnostics, Espoo, Finland; limit of detection, 1.3 nM; intra-assay CV, 3%; interassay CV, 7%).

*Acknowledgments*

Financial support was received from the Swedish Research Council (K2010-54X-09894-19-3, 2006-3832), the Swedish Foundation for Strategic Research, the ALF/LUA research grant in Gothenburg, the Lundberg Foundation, the Torsten and Ragnar Söderberg's Foundation, Petrus and Augusta Hedlunds Foundation, the Västra Götaland Foundation, the Göteborg Medical Society, the Novo Nordisk foundation and the European Commission grant HEALTH-F2-2008-201865-GEFOS.

*Disclosures*

No disclosures.

***The Cardiovascular Risk in Young Finns Study (YFS)***

The YFS is a population-based follow up-study (http://med.utu.fi/cardio/youngfinnsstudy/). The first cross-sectional survey was conducted in 1980, when 3,596 Caucasian subjects aged 3-18 years participated. The 21-year follow-up study was conducted in 2001 (ages 24-39 years) with 2,283 participants. The study cohort for the present analysis comprised 871 men who had participated in 2001 and had serum testosterone and genotype measurements, as well as other risk factor and clinical data .

*Genotyping information*

The YFS samples were genotyped using the A custom-built Illumina BeadChip Human670K from 2442 YF participants (1123 males, 1319 females) including 546,677 SNPs. Genotypes were called using Illuminus clustering algorithm. In the start of QC protocol we had 2,556 samples in YF intensity file, after initial clustering we removed 2 subjects (CR<0.90), thus the main clustering include 2,554 subjects, from these 54 samples failed QC. Thus genotyping pipeline contained 2,500 subjects. From these 54 were removed due to Sanger genotyping pipeline QC criteria (i.e., duplicated samples, heterozygosity, low call rate, or Sequenom fingerprint discrepancy). After genotyping pipeline QC the following filters were applied to the remaining data: MAF 0.01, GENO 0.05, MIND 0.05, and HWE 1e-6. 3 of 2,500 individuals were removed for low genotyping (MIND > 0.05), 11,766 markers were excluded based on HWE test (p ≤ 1e-06), 7746 SNPs failed missingness test (GENO > 0.05), 34,596 SNPs failed frequency test (MAF < 0.01 ) and one individual failed gender check. None were removed by subsequent heterozygosity check. New binary files were created after removing the individual which failed the sex-check and identity-by-descent (IBD) matrix was subsequently calculated in PLINK. There were 546,770 SNPs and 2,496 individuals at this point which were utilized to generate the genome file. There were 51 pairs of individuals with pi-hat greater than 0.2. Thus, these individuals removed due to possible relatedness. One of the pair was removed using greater missingness as criteria. After final frequency and genotyping running, there was 546,677 SNPs available from sample of 2,442 YF subjects. From these genotyped autosomal SNPs, that passed quality control those SNPs that were present on HapMap were used for imputation with MACH version 1.0 (http://www.sph.umich.edu/csg/abecasis/MACH/).

*Serum Testosterone assays*

Serum testosterone concentrations were quantified from frozen serum aliquots (stored -70°C) by commercial radioimmunoassay (RIA) kits from Diagnostic Products Corporation (Los Angeles, CA). The interassay coefficients of variation for serum testosterone for high, median, and low level controls ranged from 4.5–7.2%.

*Acknowledgments*

YFS has been financially supported by the Academy of Finland (grant no. 117797, 121584 and 126925), the Social Insurance Institution of Finland, Turku University Foundation, Tampere and Turku University Hospital Medical Funds, Emil Aaltonen Foundation (T.L), Juho Vainio Foundation, Paavo Nurmi Foundation, the Tampere Tubeculosis Foundation, the Orion-Farmos Research Foundation, Finnish Foundation of Cardiovascular Research and Finnish Cultural Foundation.

*Disclosures*

No disclosures.

**References:**

1. Purcell S, Neale B, Todd-Brown K, Thomas L, Ferreira MA, et al. (2007) PLINK: a tool set for whole-genome association and population-based linkage analyses. Am J Hum Genet 81: 559-575.

2. Sinha-Hikim I, Arver S, Beall G, Shen R, Guerrero M, et al. (1998) The use of a sensitive equilibrium dialysis method for the measurement of free testosterone levels in healthy, cycling women and in human immunodeficiency virus-infected women. J Clin Endocrinol Metab 83: 1312-1318.

3. Dawber TR, Meadors GF, Moore FE, Jr. (1951) Epidemiological approaches to heart disease: the Framingham Study. Am J Public Health Nations Health 41: 279-281.

4. Feinleib M, Kannel WB, Garrison RJ, McNamara PM, Castelli WP (1975) The Framingham Offspring Study. Design and preliminary data. Prev Med 4: 518-525.

5. Kannel WB, Feinleib M, McNamara PM, Garrison RJ, Castelli WP (1979) An investigation of coronary heart disease in families. The Framingham offspring study. Am J Epidemiol 110: 281-290.

6. Splansky GL, Corey D, Yang Q, Atwood LD, Cupples LA, et al. (2007) The Third Generation Cohort of the National Heart, Lung, and Blood Institute's Framingham Heart Study: design, recruitment, and initial examination. Am J Epidemiol 165: 1328-1335.

7. Price AL, Patterson NJ, Plenge RM, Weinblatt ME, Shadick NA, et al. (2006) Principal components analysis corrects for stratification in genome-wide association studies. Nat Genet 38: 904-909.

8. Marchini J, Howie B, Myers S, McVean G, Donnelly P (2007) A new multipoint method for genome-wide association studies by imputation of genotypes. Nat Genet 39: 906-913.

9. Sattler FR, Castaneda-Sceppa C, Binder EF, Schroeder ET, Wang Y, et al. (2009) Testosterone and growth hormone improve body composition and muscle performance in older men. J Clin Endocrinol Metab 94: 1991-2001.

10. Sir-Petermann T, Codner E, Perez V, Echiburu B, Maliqueo M, et al. (2009) Metabolic and reproductive features before and during puberty in daughters of women with polycystic ovary syndrome. J Clin Endocrinol Metab 94: 1923-1930.

11. Vesper HW, Bhasin S, Wang C, Tai SS, Dodge LA, et al. (2009) Interlaboratory comparison study of serum total testosterone [corrected] measurements performed by mass spectrometry methods. Steroids 74: 498-503.

12. Bhasin S, Storer TW, Javanbakht M, Berman N, Yarasheski KE, et al. (2000) Testosterone replacement and resistance exercise in HIV-infected men with weight loss and low testosterone levels. Jama 283: 763-770.

13. Bhasin S, Woodhouse L, Casaburi R, Singh AB, Mac RP, et al. (2005) Older men are as responsive as young men to the anabolic effects of graded doses of testosterone on the skeletal muscle. J Clin Endocrinol Metab 90: 678-688.

14. Mazer NA (2009) A novel spreadsheet method for calculating the free serum concentrations of testosterone, dihydrotestosterone, estradiol, estrone and cortisol: with illustrative examples from male and female populations. Steroids 74: 512-519.

15. Volzke H, Alte D, Schmidt CO, Radke D, Lorbeer R, et al. (2011) Cohort profile: the study of health in pomerania. Int J Epidemiol 40: 294-307.

16. Friedrich N, Volzke H, Rosskopf D, Steveling A, Krebs A, et al. (2008) Reference ranges for serum dehydroepiandrosterone sulfate and testosterone in adult men. J Androl 29: 610-617.

17. Lorentzon M, Swanson C, Andersson N, Mellstrom D, Ohlsson C (2005) Free testosterone is a positive, whereas free estradiol is a negative, predictor of cortical bone size in young Swedish men: the GOOD study. J Bone Miner Res 20: 1334-1341.

18. Estrada K, Abuseiris A, Grosveld FG, Uitterlinden AG, Knoch TA, et al. (2009) GRIMP: a web- and grid-based tool for high-speed analysis of large-scale genome-wide association using imputed data. Bioinformatics 25: 2750-2752.

19. Vandenput L, Labrie F, Mellstrom D, Swanson C, Knutsson T, et al. (2007) Serum levels of specific glucuronidated androgen metabolites predict BMD and prostate volume in elderly men. J Bone Miner Res 22: 220-227.

20. Wichmann HE, Gieger C, Illig T (2005) KORA-gen--resource for population genetics, controls and a broad spectrum of disease phenotypes. Gesundheitswesen 67 Suppl 1: S26-30.

21. Yaffe K, Barnes D, Lindquist K, Cauley J, Simonsick EM, et al. (2007) Endogenous sex hormone levels and risk of cognitive decline in an older biracial cohort. Neurobiol Aging 28: 171-178.

22. Schaap LA, Pluijm SM, Deeg DJ, Penninx BW, Nicklas BJ, et al. (2008) Low testosterone levels and decline in physical performance and muscle strength in older men: findings from two prospective cohort studies. Clin Endocrinol (Oxf) 68: 42-50.

23. Hofman A, Breteler MM, van Duijn CM, Krestin GP, Pols HA, et al. (2007) The Rotterdam Study: objectives and design update. Eur J Epidemiol 22: 819-829.

24. Ferrucci L, Bandinelli S, Benvenuti E, Di Iorio A, Macchi C, et al. (2000) Subsystems contributing to the decline in ability to walk: bridging the gap between epidemiology and geriatric practice in the InCHIANTI study. J Am Geriatr Soc 48: 1618-1625.

25. Epstein MP, Duren WL, Boehnke M (2000) Improved inference of relationship for pairs of individuals. Am J Hum Genet 67: 1219-1231.

26. Lee DM, O'Neill TW, Pye SR, Silman AJ, Finn JD, et al. (2009) The European Male Ageing Study (EMAS): design, methods and recruitment. Int J Androl 32: 11-24.

27. Labrie F, Belanger A, Belanger P, Berube R, Martel C, et al. (2006) Androgen glucuronides, instead of testosterone, as the new markers of androgenic activity in women. J Steroid Biochem Mol Biol 99: 182-188.

28. Labrie F, Belanger A, Belanger P, Berube R, Martel C, et al. (2007) Metabolism of DHEA in postmenopausal women following percutaneous administration. J Steroid Biochem Mol Biol 103: 178-188.

29. Mellstrom D, Johnell O, Ljunggren O, Eriksson AL, Lorentzon M, et al. (2006) Free testosterone is an independent predictor of BMD and prevalent fractures in elderly men: MrOS Sweden. J Bone Miner Res 21: 529-535.

30. Raitakari OT, Juonala M, Ronnemaa T, Keltikangas-Jarvinen L, Rasanen L, et al. (2008) Cohort profile: the cardiovascular risk in Young Finns Study. Int J Epidemiol 37: 1220-1226.
